# BMP-dependent, injury-induced stem cell niche as a mechanism of heterotopic ossification

**DOI:** 10.1186/s13287-018-1107-7

**Published:** 2019-01-11

**Authors:** Chen Kan, Na Ding, Jiazhao Yang, Zhenya Tan, Tammy L. McGuire, Haimei Lu, Keqin Zhang, Diana M. Palila Berger, John A. Kessler, Lixin Kan

**Affiliations:** 1 School of Basic Medical Sciences, Anhui Medical University, 81 Meishan road, Hefei, 230032 China; 2 Department of Traumatic Orthopedics, Anhui Provincial Hospital, The first Affiliated Hospital of China University of Science and Technology, Hefei, 233000 China; 3 Department of Neurology, Northwestern University, Ward Building 10-233, 303 East Chicago Avenue, Chicago, IL 60611-3008 USA; 4 Department of Endocrinology and Metabolism, Tongji Hospital, Tongji University School of Medicine, Shanghai, China; 5 Center for Comparative Medicine, Northwestern University Feinberg Medical School, Chicago, IL 60611-3008 USA

**Keywords:** Heterotopic ossification (HO), Niche, Mesenchymal stem cells (MSCs), Niche supportive cells, Niche supportive molecules, Bone morphogenetic protein (BMP), Hedgehog (Hh)

## Abstract

**Background:**

Heterotopic ossification (HO), either acquired (aHO) or hereditary, such as fibrodysplasia ossificans progressiva (FOP), is a serious condition without effective treatment. Understanding of the core process of injury-induced HO is still severely limited.

**Methods:**

Double-pulse thymidine analog labeling was used to explore the distinctive domains evolved in injury-induced lesions in an animal model of HO (Nse-BMP4). Histological studies were performed to see whether a similar zonal pattern is also consistently found in biopsies from patients with aHO and FOP. In vivo clonal analysis with Rainbow mice, genetic loss-of-function studies with diphtheria toxin A (DTA)-mediated depletion and lineage tracing with Zsgreen reporter mice were used to obtain further evidence that Tie2-cre-, Gli1-creERT-, and Glast-creERT-labeled cells contribute to HO as niche-dwelling progenitor/stem cells. Immunohistochemistry was used to test whether vasculature, neurites, macrophages, and mast cells are closely associated with the proposed niche and thus are possible candidate niche supportive cells. Similar methods also were employed to further understand the signaling pathways that regulate the niche and the resultant HO.

**Results:**

We found that distinctive domains evolved in injury-induced lesions, including, from outside-in, a mesenchymal stem cell (MSC) niche, a transient domain and an inner differentiated core in an animal model of HO (Nse-BMP4). A similar zonal structure was found in patients with aHO and FOP. In vivo clonal analysis with Rainbow mice and genetic loss-of-function studies with DTA provided evidence that Tie2-cre-, Gli1-creERT-, and Glast-creERT-labeled cells contribute to HO as niche-dwelling progenitor/stem cells; consistently, vasculature, neurites, macrophages, and mast cells are closely associated with the proposed niche and thus are possible candidate niche supportive cells. Further mechanistic study found that BMP and hedgehog (Hh) signaling co-regulate the niche and the resultant HO.

**Conclusions:**

Available data provide evidence of a potential core mechanism in which multiple disease-specific cellular and extracellular molecular elements form a unique local microenvironment, i.e., an injury-induced stem cell niche, which regulates the proliferation and osteogenic differentiation of mesenchymal stem cells (MSCs). The implication for HO is that therapeutic approaches must consider several different disease specific factors as parts of a functional unit, instead of treating one factor at a time.

**Electronic supplementary material:**

The online version of this article (10.1186/s13287-018-1107-7) contains supplementary material, which is available to authorized users.

## Background

Heterotopic ossification (HO), acquired or hereditary, is the formation of true bone in extraskeletal soft tissue [1–3]. Acquired HO (aHO) is usually associated with musculoskeletal trauma, spinal cord injury, or central nervous system injury [4], whereas hereditary HO, such as fibrodysplasia ossificans progressiva (FOP) [5], is caused by rare genetic mutations. Regardless, all forms of HO are serious medical conditions that are often closely associated with local abnormal injury/inflammatory responses (AIR) [3]. HO forms mainly through a process called intracartilaginous endochondral ossification (or endochondral ossification), meaning that ossification is preceded by cartilage, similar to the bones of the limbs. However, in the context of injury-induced HO, cartilage formation is preceded by a series of stereotyped sequential events, i.e., early injury-induced inflammation (inflammatory stage), fibroproliferation (fibroproliferative stage), and condensation (condensation stage) [1]. The specific pathological mechanisms leading to HO are still largely unknown, although many different factors have been implicated in the process [6–9].

Based on long-term observations, we hypothesized that multiple disease-specific cellular and extracellular molecular elements form a unique and relatively stable local microenvironment, i.e., an injury-induced mesenchymal stem cell niche (MSC niche), which regulates the proliferation and subsequent osteogenic differentiation of mesenchymal stem cells (MSCs) and the downstream HO process. This study was designed to directly test this hypothesis. One commonly accepted approach for identifying and localizing stem cells in the niche is through the label-retaining cell (LRC) technique, which normally depends on administration of a marker that is incorporated into the DNA of proliferating cells and retained by stem cells for long periods of time after the labeling period because of the slow-cycling, quiescent nature of stem cells. To maximize the temporal-spatial resolution of the LRCs, we used a dual-pulse CldU and IdU labeling (dual LRC) protocol [10] at different stages of the HO process in this study.

We also took advantage of the high spatial-temporal resolution afforded by Rainbow reporter mice to provide a way for in vivo clonal analyses of stem cells that contribute to HO. The R26R-Confetti allele in these mice acts as a stochastic multicolor Cre recombinase reporter of multiple fluorescent proteins from a single genomic locus [11]. Previous studies have identified a number of populations of cells that contribute to HO including Tie2, Glast, and Gli1-expressing mesenchymal stem/progenitor cells (MSCs) [7–10], and validated Tie2-cre [12, 13], Glast-creERT [14], and Gli1-creERT [15] mouse lines are commercially available. These Cre lines allowed us to establish the functional role of these different populations of cells using Cre-induced diphtheria toxin A (DTA) expression to specifically deplete individual target MSC subpopulations [16].

Numerous prior studies have implicated various signaling pathways, such as bone morphogenetic protein (BMP) BMP, wnt/β-catenin, fibroblast growth factor (FGF), hedgehog (Hh) signaling, and probably other conserved pathways that participate in the HO process [17]. The BMP signaling pathway is the most extensively studied one, and it is generally accepted that BMP signaling is crucial for the osteogenic and chondrogenic processes [18]. There also is evidence that implicates Hh and wnt/β-cat signaling in HO [19]; however, it is still largely unknown how these signaling pathways interact and co-regulate the HO process, or whether these signaling pathways play roles in the proposed MSC niche. The tools that we developed allowed us to explore the role of these signaling molecules in regulating MSC proliferation and differentiation in HO lesions.

Overall, this proof-of-concept study provides evidence of a potential core mechanism in which multiple disease-specific cellular and extracellular molecular elements form a unique local microenvironment, i.e., a MSC niche, where BMP and Hh signaling co-regulate this process. The insights gained from this study have broad implications not only for prevention and treatment of HO, but also for potential translational applications of MSCs in a variety of injury or regenerative-related disorders.

## Materials and methods

### Animals and injury models

The Nse-BMP4 transgenic mice used in this study have been described previously [14, 20]. This line overexpresses bone morphogenetic protein 4 (BMP4) under the control of neuron-specific enolase (Nse) promoter and develops injury-induced HO robustly. The Gli1^tm3(cre/ERT2)Alj^/J (Gli1-creERT) [21], Tg(Slc1a3-cre/ERT)1Nat/J (Glast-creERT), B6.Cg-Tg(Tek-cre)1Ywa/J (Tie2-cre) [22], Gt(ROSA)26Sor^tm1(DTA)Jpmb/J^ (ROSA26-eGFP-DTA) [16], B6.129P2-Gt(ROSA)26Sor^tm1(CAG-Brainbow2.1)Cle/J^ (R26R-Confetti, also called Rainbow mice) [23], and the B6.Cg-Gt(ROSA)26Sor^tm6(CAG-ZsGreen1)Hze^/J) lines [24] (Zsgreen reporter) were from the Jackson Laboratory (Bar Harbor, ME). To conditionally induce Gli1-creERT or Glast-creERT expression, 100 μl of tamoxifen (TAM, 10 mg/ml) was injected intraperitoneally into adult (> 1 month) mice as previously reported [14].

The Zsgreen reporter mouse has a loxP-flanked STOP cassette that prevents transcription of the downstream enhanced green fluorescent protein (ZsGreen1) in the absence of Cre. However, when bred to mice that express Cre, the STOP cassette is permanently deleted in Cre-expressing cells resulting in constitutive expression of ZsGreen1 in those cells and all of their progeny. This is a gold standard approach for lineage tracing; however, Zsgreen reporter lines, or other unicolored reporter lines, are generally not suitable for clonal analysis, since the clonal resolution is too low, especially in long-term tracing.

R26R-Confetti (Rainbow) is a reporter line that, when bred to mice that express Cre recombinase, generate offspring with a recombination event that stochastically places one of the four fluorescent proteins into a position directly downstream of the CAG promoter within the Cre-expressing tissues. The random nature of this recombination event significantly increases the clonal resolution and therefore provides a high-resolution way to follow the progeny of individual stem/progenitor cells (i.e., clonal analysis) in vivo [23]. The caveat is that, due to a variety of potential limitations, not all recombination events can be detected unequivocally with comparable efficiency, which often leads to underestimation, or even severe underestimation, of recombination efficiency; fortunately, for clonal analysis, relatively low detection efficiency is actually beneficial.

ROSA26-eGFP-DTA is a mouse line useful for conditional depletion of specific subpopulations of cells. Once bred to mice that express Cre recombinase under the control of a promoter of interest, the loxP-flanked EGFP and stop sequence are removed, and diphtheria toxin (DTA) expression is activated, resulting in the specific ablation of Cre-expressing cells [16]. The deletion efficiency can be estimated by calculating the ratio of GFP^−^ (recombined)/GFP^+^ cell population (unrecombined).

The standard muscle injury procedure was performed according to previous reports [14, 20]. Adult mice, both males and females, were used in this study. All animals were randomly allocated to groups that each contained at least five mice at each time point. All mice were house in standard (pathogen free) conditions and were fed with standard chow (Irradiated Teklad LM-485 Mouse/Rat Diet, Catalog# 7912.15 or Catalog# 7904.15, Envigo), ad libitum. In all experiments, the investigators performed all analyses blinded to the identity of the animals. For each mouse (either from control or experimental groups), at least 10 random sections were obtained through the lesions. All animal experiments in this study were approved by the Animal Care and Use Committees at Anhui Medical University and at Northwestern University.

### Patients and clinical data summaries

We retrospectively examined the histological features both in FOP (3 patients) and aHO (8 patients) (see Additional file 1: Table S1 for overall summary of clinical data, Additional file 2: Table S2 for summary of the histomorphometric analysis, Additional file 3: Figure S1 for the gross HO images and Additional file 4: Figure S2 for the typical histological features). The biopsies of the lesional tissues from patients of FOP were obtained as a diagnostic test before a clear FOP diagnosis being established. The biopsies from patients of aHO were also obtained as a part of diagnostic test/resection surgery. The histologic data from these samples were systematically studied to identify the target issues (the three domain structure, i.e., the MSC niche, transient domain and inner differentiated core), based on typical morphological features. For each individual patient, at least five or more H&E images were reviewed and analyzed. Generally, the target issues are located on the periphery of lesional samples from the condensation stage onward; however, some samples did not contain significant amounts of the target tissues due to temporal (sampling too early or too late) and/or spatial (does not contain the periphery of the lesions) limitations (Additional file 1: Table S1). The presented FOP data was from a 7-year-old girl whose chief complaint was widespread unknown swelling with restricted joint motion of the upper arm. The FOP diagnosis was established later, based on histological findings and the typical deformity of the great toe, i.e., hallux valgus and shortened great toe. The presented aHO data was from a 56-year-old male with a chief complaint of limited right hip joint motion due to a total hip arthroplasty surgery. This retrospective study used pre-existing fully deidentified clinical samples and data, and this study was approved by the ethics committee of Anhui provincial Hospital, The First Affiliated Hospital of China University of Science and Technology.

### In vivo clonal analyses and DTA-mediated ablation of cells

To perform clonal analyses, we first generated triple transgenic mice, i.e., Nse-BMP4;Gli1-creERT;R26R-Confetti, Nse-BMP4;Glast-creERT;R26R-Confetti, and Nse-BMP4;Tie2-cre;R26R-Confetti, respectively. We then traced the labeled cells at different time points after TAM induction and injury, using standard procedures [25], except that Tie2-cre is not inducible. Briefly, we determined cell clusters by measuring the distance between the cells labeled with the same color through ImageJ. Specifically, if the neighboring labeled cells were of the same color (uninterrupted by cells with a different color) and within a distance of about up 100 μm with each other (between two nearest points of neighboring cells), these cells were considered of the same simple cluster, but the clusters with less than three cells were not treated as an independent clone (i.e., cutoff value = 3). The distance of 100 μm was chosen based on our preliminary studies and consideration of several crucial factors: (1) degree of dispersion of individual colonies, (2) the labeling efficiency with each Cre line, and (3) minimization of both false positivity and false negativity. In our model, the degree of dispersion of individual colonies is relatively large, and the labeling efficiency with R26R-Confetti is generally low, which led to the criteria that were then applied prospectively to all groups in this study. Under some conditions, neighboring clusters overlapped (i.e., some cells were located in the middle of two or more potentially independent clones). In this case, none were treated as independent clones, which unavoidably reduced the clonal resolution. To quantify the clonal resolution, we counted the number of the labeled cells within an independent cluster and the total number of labeled cells in the same field, and the clonal resolution = the ratio of the number of the labeled cells within an independent cluster/total number of labeled cells in the same field. The labeling efficiency = the ratio of the number of all labeled cells (from all different channels)/total cells in the same field. The labeling efficiency is influenced by a variety of different factors, such as the expression profile of different Cre lines, the length and intensity of TAM induction, the recombination efficiency of the specific Cre, and the detection efficiency.

To study the functional consequences of conditional depletion of Tie2^+^, Glast^+^, or Gli1^+^ stem/progenitor cells, we generated additional sets of triple transgenic mice, i.e., Nse-BMP4;Tie2-cre;ROSA26-eGFP-DTA, Nse-BMP4;Gli1-creERT;ROSA26-eGFP-DTA, and Nse-BMP4;Glast-creERT;ROSA26-eGFP-DTA. TAM was administered as described above. To gauge the effects of target cell depletion on the proposed MSC niche, we measured the average thickness of the MSC niche based on the images of the lesion from the TAM-treated and control animals (without TAM). Specifically, we took images around the lesional core, i.e., we took four pictures/differentiated core (one picture from each quadrant) for each mouse (five mice in each group) and then measured in a blinded fashion the average thickness of the MSC niche in each image based on the abovementioned morphological features. To quantify the depletion efficiency in this model, we calculated the ratio of GFP^−^ cells in treated and control animals/total cells in the same target areas.

Histomorphometric analysis was performed with software Image Pro plus 6.0, IPP 6.0 (Media Cybernetics Inc., Silver Spring, MD, USA). Several parameters were included: (1) Trabecular area/bone area is the percentage of area occupied by calcified bone in relation to the total area. (2) Trabecular width is the distance across individual trabeculae = (2× bone area)/bone perimeter). (3) Trabecular number is the number of trabeculae that a line through a trabecular compartment would hit per millimeter of its length = (bone area/trabecular area)/trabecular width. (4) Trabecular separation is the distance between trabeculae = (1/trabecular number) − trabecular width. (5) Niche area is the total area that matches the criteria of the MSC niche (right location, with typical fibrotic cell morphology).

### Cldu/Idu double-pulse staining

On the day of injury, CldU (42.5 mg/kg) was injected intraperitoneally into adult (> 1 month old) Nse-BMP4 mice three times per day. One day before sacrifice, two IdU (57.5 mg/ kg) injections were administered. The animals were sacrificed and the tissues were harvested at different time points. To double stain CldU and IdU, sections were first denatured by incubating the sections in 2 N HCl for 30 min at 37 °C, neutralized with 0.1 M sodium borate, pH 8.5, for 10 min at room temperature, rinsed twice with PBS, and incubated with blocking solution (10% normal goat serum) at room temperature for 1 h. The sections were then incubated with primary antibodies diluted with 1% BSA + 0.25% Triton X-100 at 4 °C overnight. The sections were then incubated with appropriate secondary antibodies (Alexa Fluor 488, Alexa Fluor 594, or Alexa 647 conjugated antibodies diluted with 1% BSA + 0.25% Triton X-100 in the dark at room temperature for 2 h). Counterstaining was then performed with 4,6-diamidino-2-phenylindole (1:5000). To quantify the distribution of different LRC subpopulations, we counted the CldU^+^, IdU/CldU^+^, and IdU^+^ cells in different domains, including the proposed MSC niche, the transient domain, and the inner differentiated core, respectively. The overall quantification was based on 75 high power (40×) images (=5 animals × 3 sections × 5 images).

### Other immunostains

Immunostaining for different markers was performed as previously described [14, 20]. Briefly, sections were pre-fixed with 4% paraformaldehyde in PBS. Nonspecific binding was blocked with 10% normal serum diluted in 1% bovine serum albumin (BSA; Jackson ImmunoResearch Laboratories, West Grove, PA) and 0.25% Triton X-100 (Sigma) for 1 h in room temperature. Thereafter the steps were the same as described above for staining of CldU and IdU.

Primary antibodies against CldU (Rat, Bio-Rad, #OBT0030CX), IdU (mIgG1, BD, 347580), S100A4 (mIgG1, Novus, 1F12-1G7), STRO1 (mIgM, Santa Cruz, sc-47733), endomucin (Rat, sc-65495, Santa Cruz), SMI312 (Covance, # SMI-312R), TRY (Abcam, Anti-Mast Cell Tryptase antibody [AA1] (ab2378)), IBA1(Dako, S3022), collagen IV (Abcam, ab6586), collagen VI (Abcam, ab182744), tenascin C (Rat, SIGMA, clone MTn-12, T3413), laminin (SIGMA, L9393), chondroitin sulfate proteoglycan (IgM, SIGMA, C8035), BMP4 (Abcam, ab39973), BMPRII (Abgent, BMPR2 antibody (N-term), Q13873), and pSMAD1/5 (MilliporeSigma, anti-phospho Smad1/Smad5/Smad8 (Ser463/465), AB3848-I) were used in this study. The specificity of all antibodies was established by comparing the specific staining signal with negative control (without 1st Ab) and/or in the presence of blocking peptide (not shown).

## Results

### Validation of the dual-pulse CldU and IdU labeling procedure

The label-retaining cell (LRC) technique is a commonly accepted approach to identify different stem cell populations. To maximize the temporal-spatial resolution, we used a dual-pulse CldU and IdU labeling (dual LRC) protocol [10]. To validate this procedure in our model, we used two positive control tissues, i.e., intestinal mucosa (jejunum) and hair follicles (skin), that each has large, well-characterized populations of tissue-specific adult stem cells (LRC^+^). For the negative control, we used uninjured adult muscle in which the turnover rate is minimal. As expected, we observed abundant specific staining in the positive controls including CldU^+^IdU^−^ (long-term/quiescent stem cells, red arrows), CldU^+^/IdU^+^ (short-term/active stem cells, yellow arrows). and CldU^−^IdU^+^ (transient amplifying cells, green arrows) cells (Additional file 5: Figure S3A & B). In contrast, in uninjured adult muscle, no appreciable specific staining was observed (Additional file 5: Figure S3C & D). Additional file 5: Figure S3E is the schematic paradigm of the dual-pulse CldU and IdU labeling.

### The stereotyped patterns (domains) of LRCs in developing HO suggest the existence of a stem niche

We then examined the patterns of LRCs in different stages of HO, using the established dual-pulse labeling procedure. The condensation stage (about 6 days after injury) divides the HO process into early and late HO stages, i.e., stages earlier than the condensation are generally referred to as “early”, while stages later than the condensation are referred to as “late”. Notably, the definite HO process and the MSC niche begin to form from the condensation stage onward. The rationale for this staging is provided in our prior publications [7, 8, 20]. At each time point (stage), at least 5 mice were examined and at least 10 random sections through the lesion were obtained from each mouse. The quantification of CldU^+^ and IdU^+^ cells at each time point was based on CldU^+^/IdU^+^ double staining in a minimum of 25 sections (5 mice × 5 images/stage).

**Fig. 1:**
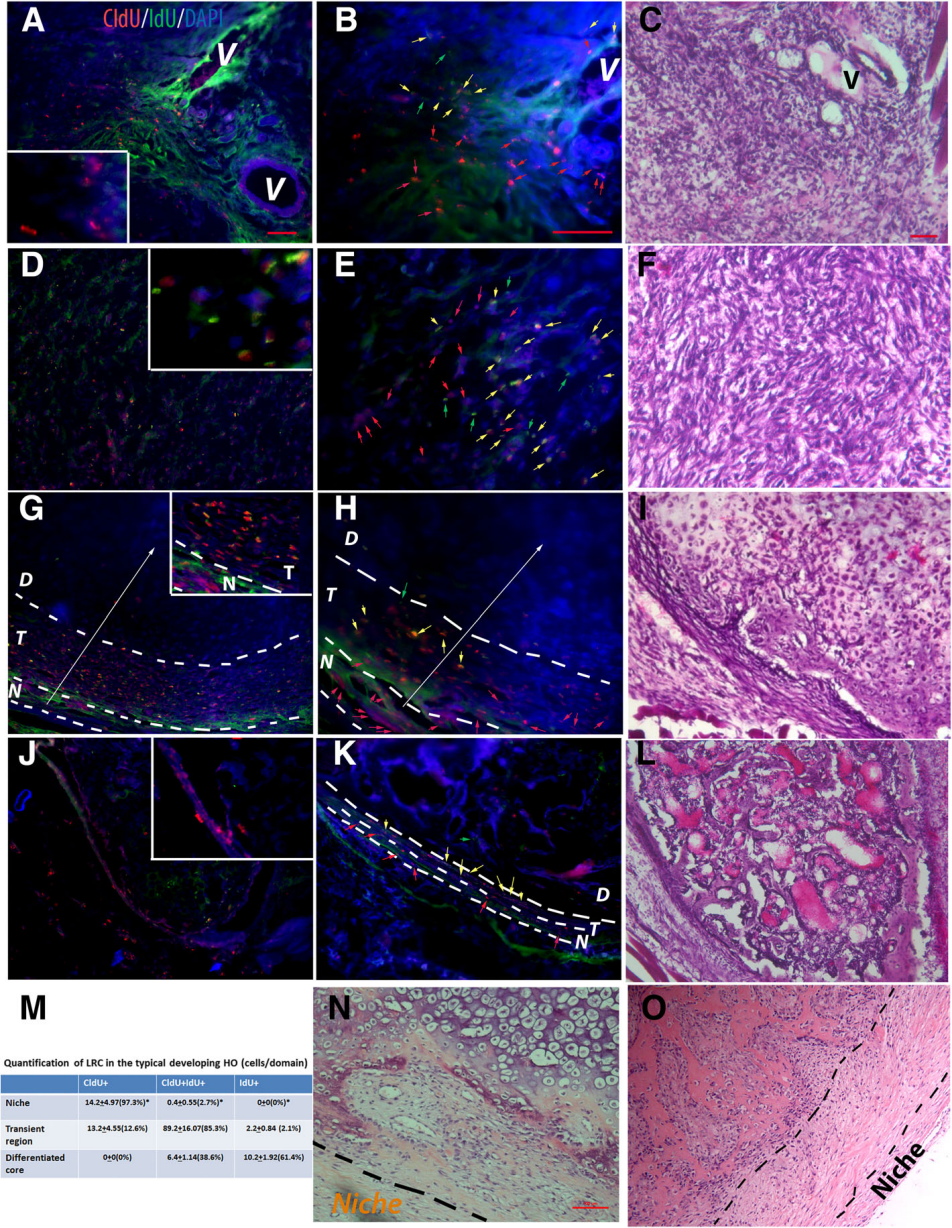
The patterns of LRCs in different stages of HO. **a–c** In the early inflammatory stage (< 3 days after injury) (**a** low power, **b** high power, and **c** H&E staining of the adjacent section), typical images showed many CldU^+^/IdU^−^ cells (quiescent stem cells, red arrows), some CldU^+^/IdU^+^ (active stem cells, yellow arrows), but very few CldU^−^/IdU^+^ (transient amplifying cells, green arrows). Inserted box is a partial enlarged target area of panel **a** that highlights the staining pattern at this time point; note that no recognized regional pattern can be identified. **d–f** In the fibroproliferative stage (3–6 days after injury) (**d** low power, **e** high power, and **f** H&E staining of the adjacent section), typical images showed few CldU^+^/IdU^−^ cells (red arrows), many CldU^+^/IdU^+^ (yellow arrows), and some CldU^−^/IdU^+^ (green arrows). Inserted box is a partial enlarged target area of panel **d** that highlights the staining pattern at this time point; note that no recognized regional pattern can be identified. **g–i** In developing HO (> 6 days after injury) (**g** low power, **h** high power, and **i** H&E staining of the adjacent section), the lesion could be roughly divided based on the consistent LRC pattern into three adjacent domains with a perceivable gradient from outside to the middle of the lesion (white arrows), i.e., the MSC niche (*N*), the transient domain (*T*), and the inner differentiated core (*D*). Inserted box is a partial enlarged target area of panel **g** that highlights the niche and the adjacent transient region; note the significant difference of staining pattern in these two different regions. (Also note the diffuse non-nuclei green staining is the background signal). Histologically, the MSC niche (*N* in **g** and **h**) was a very narrow strip. LRCs in this domain were almost exclusively CldU^+^/IdU^−^ cells (red arrows). The transient amplifying domain (*T* in **g** and **h**), that was located between the MSC niche and the inner differentiated core, contained both CldU^+^/IdU^+^ (active stem cells, yellow arrows) and CldU^−^/IdU^+^ (transient amplifying cells, green arrows), with a perceivable gradient (white arrow), and the inner differentiated core (*D* in **g** and **h**) was composed of differentiated cells without significant thymidine analog label. **j–l** In mature HO (> 1 month after injury) (**j** low power, **k** high power, and **l** H&E staining of the adjacent section), the lesion could be similarly divided into three adjacent domains, except that the transient domain was often absent in the mature HO. Inserted box is a partial enlarged target area of panel **j** that highlights the narrow niche that contains almost exclusive the quiescent CldU^+^/IdU^−^ cells and the adjacent region. **m** Quantification of different types of LRC in different domains of developing HO (2 weeks after injury). Average cell number/domain area in high-power image ± SD was shown (*n* = 5); percentages in parentheses represent the percentages of different subpopulations (CldU^+^, CldU^+^/IdU^+^, or IdU^+^) over the total LRC^+^ cells within each domain); statistics compared the percentages of positive cells in MSC domain vs. other domains, *Differ at *p* < 0.05 by ANOVA with Bonferroni post hoc testing. Note that in the proposed niche, 97.3% of total LRCs are CldU^+^ quiescent stem cells. In contract, in proposed transient region, most (89.2%) of LRCs are CldU^+^/IdU^+^ active stem cells, and in terminal differentiated core, the majority (61.4%) of LRCs are IdU^+^ transient amplifying cells. **n**, **o** A similar zonal pattern could be identified in human FOP (**n**) and aHO (**o**) samples, based on cell morphology (typical images of H&E staining shown). **a**, **d**, **g**, and **j** are on the same scale; **b**, **e**, **h**, and **k** are on the same scale; **c**, **f**, **i**, and **l** are on the same scale, bar = 50 μm. **n** and **o** are on the same scale, bar = 100 μm

In early inflammatory lesions (< 3 days after injury), we found many quiescent stem cells (CldU^+^IdU^−^, red arrows), some active stem cells (CldU^+^/IdU^+^, yellow arrows), but very few transient amplifying cells (CldU^−^/IdU^+^, green arrows) (Fig. 1a–c). In fibro-proliferative stages (3–6 days after injury) (Fig. 1d–f), we found fewer quiescent stem cells, many active stem cells, and some transient amplifying cells, which suggested the activation of stem cells or shift of the proliferative status of stem/progenitor cells within these two pre-HO stages. However, there was no consistent distribution pattern of LRCs in early pre-HO stages, or easily recognized morphological features (typical elongated fibroblast-like morphology was not consistently observed until well-developed fibro-proliferative stages).

In contrast, there was an easily recognizable zonal pattern of LRCs from the condensation stage onward (> 6 days after injury) (Fig. 1g–l). Specifically, based on LRC staining, three adjacent domains could be identified in typical developing HO lesions, i.e., from outside-in, the niche proper, a transient domain, and an inner differentiated core. Each domain had its own unique features. For example, the proposed niche (i.e., *N* in Fig. 1g, h, and k) was a very narrow strip (no more than a few cells in thickness) that contained almost exclusively CldU^+^/IdU^−^ cells (about 97.3% of LRC in this domain)(*red arrows*), which strongly supported our hypothesis. Histologically, most cells in the niche proper showed an elongated fibroblast-like morphology. The transient domain (*T* in Fig. 1g, h, and k) was located between the niche proper and the inner differentiated core. This somewhat variable domain contained few quiescent stem cells (about 12.6% of LRC in this domain) (red arrows), many active stem cells (about 85.3% of LRC in this domain) (yellow arrows) and rare transient amplifying cells (about 2.1% of LRC in this domain) (green arrows), with a perceivable gradient (white arrow), i.e., the closer to the center of the lesion, the fewer LRC (less stemness). Consistently, the morphology of the cells in the transient domain also reflected this progressive pattern. The inner differentiated core (*D* in Fig. 1g, h, and k) was mostly composed of unlabeled differentiated cells with the typical morphology of chondrocytes or hypertrophic chondrocytes. Only a few LRC+ cells were scattered in this domain. As expected, most of the LRC^+^ cell in this domain were not stem cells (CldU^−^/IdU^+^, about 61.4% of LRC in this domain) (Fig. 1h and m). Similar features also were found in mature HO (> 1 month after injury) (Fig. 1j–l), except with a much narrower transient domain (the demarcation between the niche proper and the transient domain could be indistinct at this stage) and a well-developed mature HO core with trabeculae and bone marrow.

We then retrospectively examined the samples from patients of FOP and aHO (see Additional file 1: Table S1 for overall summary of clinical data, Additional file 2 Table S2 for summary of the histomorphometric analysis, Additional file 3: Figure S1 for the gross HO images and Additional file 4: Figure S2 for the typical histological features). Importantly, the typical zonal pattern was identified in all samples that contain target tissues, both in FOP (Fig. 1n) and aHO (Fig. 1o) samples, though some sections did not contain enough target tissues to make a definite judgment (see Additional file 1: Table S1 and Additional file 2: Table S2 for details). This zonal pattern mimics stem cell niches in other organs, such as the neural stem cell niche in adult brain, suggesting the existence of an injury-induced stem cell niche. Furthermore, many of the CldU^+^/IdU^−^ cells (quiescent stem cells) in the proposed niche co-labeled with the candidate MSC markers, Stro1 and S100A4 [26], in both the early inflammatory stage and in developing HO (Additional file 6: Figure S4), suggesting a MSC identity of MSC for at least a subpopulation of LRC. Partial co-localization with MSC markers was consistent with the idea that MSC, a heterogeneous adult mesenchymal stem population, could be the niche-dwelling stem cells.

### In vivo clonal analysis suggests that Tie2-cre-, Glast-creERT-, and Gli1-creERT-labeled progenitor/stem cells might be niche-dwelling stem cells and contribute to HO through the MSC niche

To further characterize how the stem/progenitor cells contribute to HO, we generated triple transgenic mice, i.e., Nse-BMP4;Gli1-creERT;R26R-Confetti, Nse-BMP4;Glast-creERT;R26R-Confetti and Nse-BMP4;Tie2-cre;R26R-Confetti, and clonally analyzed the labeled subpopulations at different stages of injury-induced HO. The underlying rationale is that the rainbow reporter mice could provide high enough clonal resolution to enable in vivo analysis of stem cell behavior; however, since prolonged subsequent migration could potentially disrupt clonal integrity, our tracing focused primarily on the early stages of HO (i.e., 1–2 weeks after injury). Similar to the LRC study, at each time point (stage), at least 5 mice were examined and at least 10 random sections through the lesion were obtained from each mouse. In addition, in a small subset (four mice), we waited 3–4 weeks to sacrifice the mice to examine subsequent events, such as the migration pattern. Overall, we found that although the labeling efficiency varied among Cre lines, and even between individual animals, cluster-like (clonally derived) Cre-labeled cells were found in all stages of HO. Importantly, all three Cre lines worked as expected outside of the target tissues (Additional file 7: Figure S5).

**Fig. 2:**
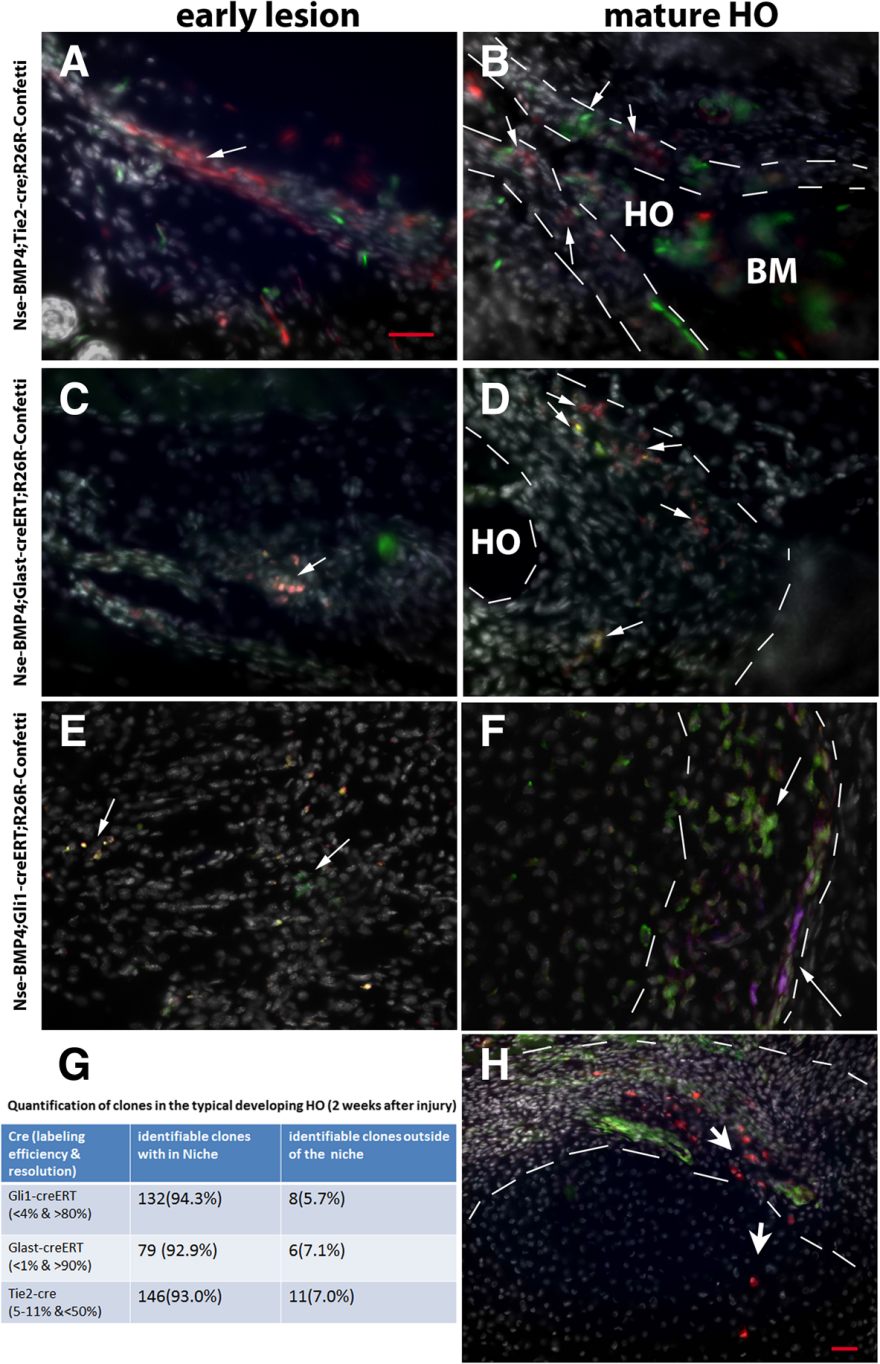
In vivo clonal analysis of subpopulations in the MSC niche in triple transgenic mice. **a**, **b** Typical images from early (**a**) and late (**b**) lesions of Nse-BMP4;Tie2-cre;R26R-Confetti mice. Note that many clonal clusters are located in the proposed MSC niche in (**b**) (between the white dashed lines). **c**, **d** Typical images from early (**c**) and late (**d**) lesions of Nse-BMP4;Gli1-creERT;R26R-Confetti mice. Note that the labeling efficiency was generally low in Gli1-creERT;R26R-Confetti mice, and the clonal clusters were easily identifiable. Similarly, the majority of clonal clusters were located in the proposed MSC niche (between the white dashed lines). **e**, **f** Typical images from early and late lesions of Nse-BMP4;Gli1-creERT;R26R-Confetti mice. Similarly, the majority of clonal clusters were located in the proposed MSC niche. White arrows point to clonal clusters in all panels. **g** Summary of in vivo clonal analysis in developing HO in each triple transgenic mice, *n* = 5 / group. **h** Typical image from late stages (3 weeks after injury) of lesions that appear to catch the migration pattern of progeny (white arrows, from MSC to inner core). **a**, **d**, **g**, and **j** are on the same scale; **b**, **e**, **h**, and **k** are on the same scale, bar = 50 μm. **n** and **o** are on the same scale, **a**–**f** are on the same scale; bar = 50 μm.

Despite these common themes, the three tested Cre lines all had their own unique features. For example, Tie2-cre;R26R-Confetti mice (Fig. 2a, b, and g) generally had the highest labeling efficiency (about 5–11%) and the lowest clonal resolution (average clonal resolution < 50%). Nevertheless, from the condensation stage onward, individual clonal clusters were still identifiable. More importantly, up to 93% of Tie2-cre-labeled clusters were in the proposed MSC niche (based on the characteristic cell morphology), reflecting the establishment of a relatively stable MSC niche in lesional tissues. In contrast, in inducible Nse-BMP4;Glast-creERT;R26R-Confetti mice (Fig. 2c, d, and g), the labeling efficiency was generally low (< 1%), and the sparser labeling made identification of clonal clusters (average clonal resolution > 90%) easier to visualize even in the early stages. The labeling efficiency with Nse-BMP4;Gli1-creERT;R26R-Confetti mice was intermediate between the other two lines (about 0.5–4%), which made identification of clonal clusters harder in early stages; nevertheless, individual clonal clusters were often identifiable (Fig. 2e, f, and g). Importantly, the overwhelming majority of clonal clusters in developing HO were found in the proposed MSC niche, which strongly supported our hypothesis (Fig. 2g). Furthermore, clonal expansion/migration apparently followed a stereotyped pattern, i.e., from niche proper towards the inner core, based on multiple tracing at different time points (Fig. 2h). This pattern suggested that niche-dwelling stem cells contribute to HO through clonal expansion followed by migration and lineage-specific differentiation as predicted by our central hypothesis.

### Conditional depletion provided further evidence that Glast-creERT- and Gli1-creERT-labeled progenitor/stem cell subpopulations contribute to the MSC niche

Clonal analysis provided evidence that the behavior of these different mesenchymal stem/progenitor subpopulations was consistent with them being niche-dwelling stem cells, but a causal relationship could not be established just by clonal analysis. We reasoned that if these subpopulations were truly the main contributing cells of the MSC niche, depletion of these populations should disrupt the MSC niche and the downstream process. To test this idea, we took a loss-of-function approach using ROSA26-eGFP-diptheria toxin (DTA) mice to induce cell death of those specific target populations of cells. We focused mainly on two relevant lines, Nse-BMP4;Glast-creERT;ROSA26-eGFP-DTA and Nse-BMP4;Gli1-creERT;ROSA26-eGFP-DTA, because the attempt to create Nse-BMP4;Tie2-cre;ROSA26-eGFP-DTA mice rarely produced offspring of desired genotype (i.e., Nse-BMP4;Tie2-cre;ROSA26-eGFP-DTA), possibly due to the potential developmental complications associated with unconditional Tie2-cre expression.

**Fig. 3:**
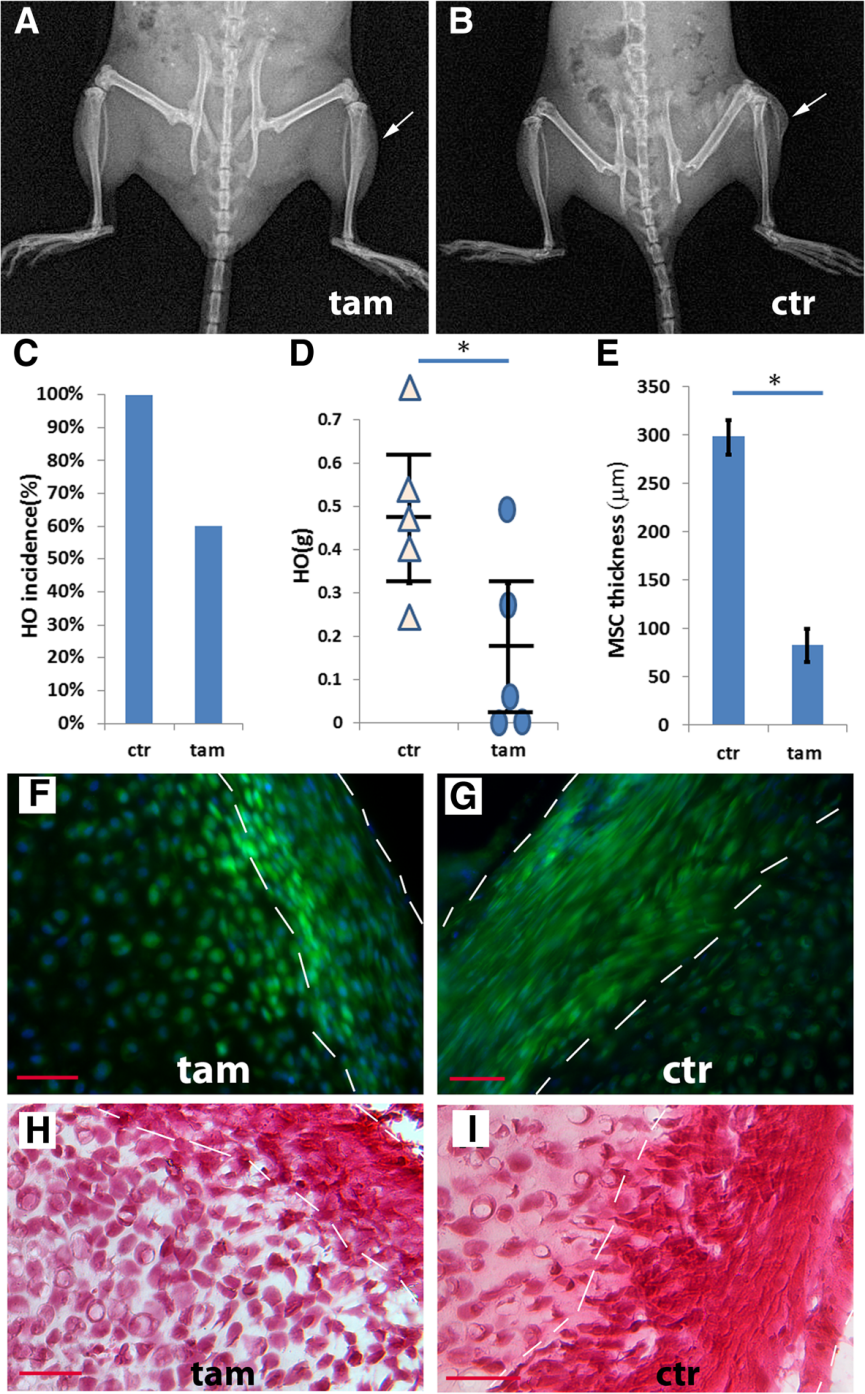
Conditional depletion of the Glast-creERT+ subpopulation inhibited injury-induced HO. **a**, **b** A typical X-ray image of TAM-treated (**a**) and control (**b**) Nse-BMP4;Glast-creERT;ROSA26-eGFP-DTA mice 4 weeks after injury. **c** HO incidence in control and TAM-treated groups. **d** Quantification of wet weight of HO in control and TAM treated groups, *n* = 5. Statistical analysis compared control (without TAM) vs. depleted (with TAM), using an unpaired *t*-test; *P < 0.05. Note that depletion of Glast-creERT^+^ cells inhibited but did not completely block HO. **e** Quantification of the thickness of MSC niche, *n* = 5, Statistical analysis compared control (without TAM) vs. depleted (with TAM), using an unpaired *t*-test; *P < 0.05. **f** Typical fluorescence images from TAM-treated (**f**) and control (**g**) Nse-BMP4;Glast-creERT;ROSA26-eGFP-DTA mice. Note that in the TAM-treated group (**f**), GFP^−^ (recombined but not depleted) cells were only rarely found. **h**, **i** H&E staining of sections from TAM-treated (**h**) and control (**i**) Nse-BMP4;Glast-creERT;ROSA26-eGFP-DTA mice. Note that both the fluorescence images and the H&E staining suggest that the proposed MSC domain (within dashed white lines) was thinner in the TAM treated group. Bar = 50 μm

TAM treatment completely blocked HO in some of the Nse-BMP4;Glast-creERT;ROSA26-eGFP-DTA mice (2/5), and partially inhibited HO in the rest (3/5) (Fig. 3a–d). Interestingly, the putative MSC niche was much thinner than (Fig. 3e) that of controls even in the mice in which HO was not completely inhibited (see Additional file 8: Table S3 for the histomorphometric analysis). To determine whether the persistence of a diminished niche reflected incomplete deletion of the target stem cell population, we counted GFP^−^ cells in HO lesions in the triple transgenic mice, with or without TAM, and found that incomplete deletion was insignificant (Fig. 3f and g and data not shown), supporting the idea that Glast-creERT-labeled population is not the only contributing population of MSC niche. Further detailed study also found that there was no obvious stage-specific phenotype (Fig. 3e–i, Additional file 8: Table S3, Additional file 9: Figure S6). Consistently, we found similar results after conditional depletion of Gli1-creERT-labeled progenitor/stem cells in Nse-BMP4;Gli1-creERT;ROSA26-eGFP-DTA mice (Additional file 10: Figure S7), suggesting that Gli1-creERT-labeled cells also contributed to HO through the MSC niche.

### Vasculature, neurites, mast cells, and macrophages are likely candidate niche supportive cells

**Fig. 4:**
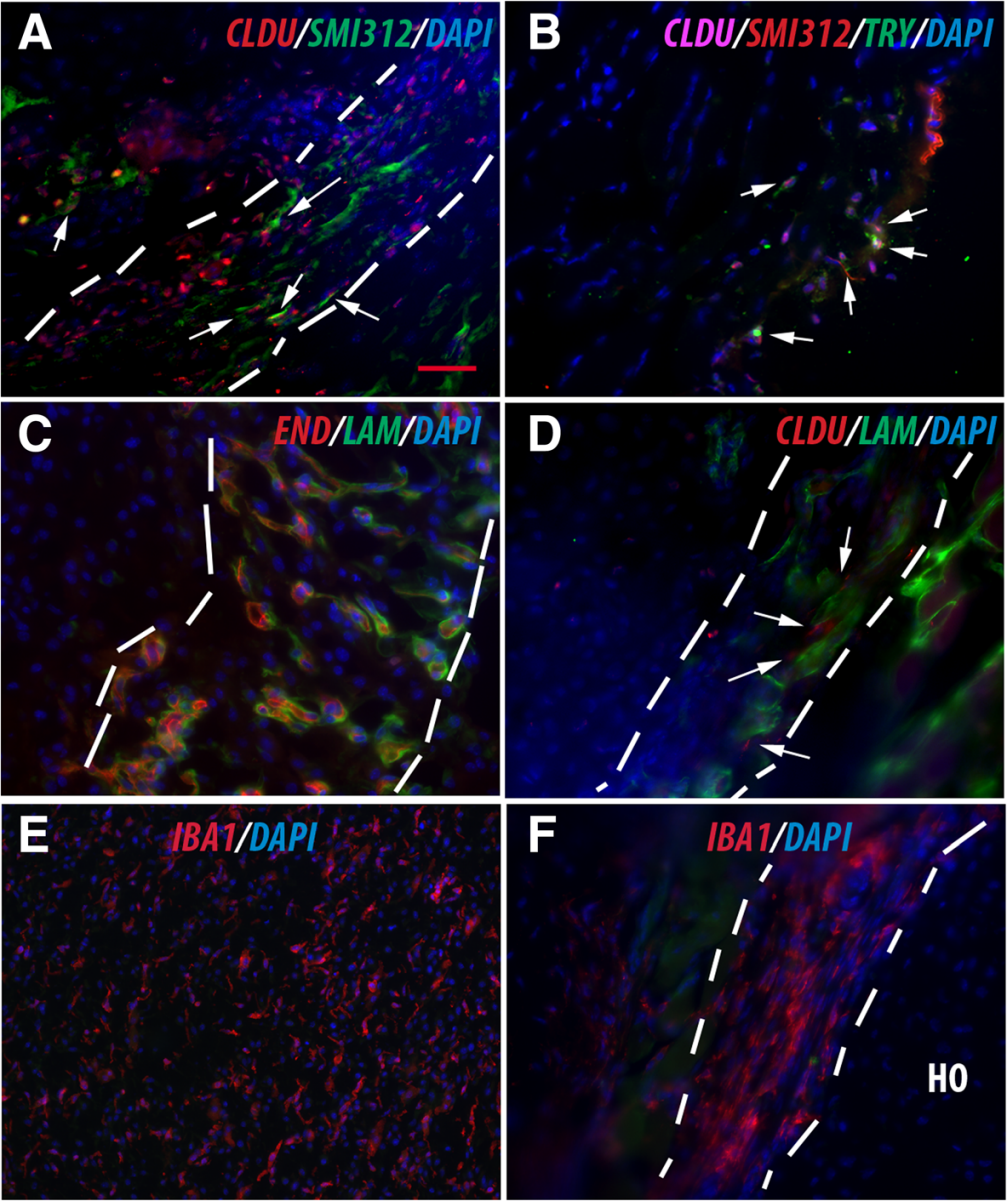
Characterization of the candidate niche supportive cells. **a** Typical image of double staining of CldU^+^/SMI312. White arrows point to the close association but not co-localization of CldU^+^ stem cells with SMI312^+^ neurites. **b** Typical image of triple staining of CldU^+^/SMI312/Typ. White arrows point to close association but not co-localization of CldU^+^ stem cells with Typ + mast cells. **c** Typical image of double staining of (end/lam). Note the almost 100% association but not co-localization of end with lam (end is always on the luminal side). **d** Typical image of double staining of (CldU/lam). White arrows point to close association but not co-localization of CldU^+^ stem cells with lam + vasculature. **e**, **f** Macrophages were associated with HO. **e** Abundant IBA1^+^ macrophages were present in the lesion from the earliest stage. **f** IBA1^+^ macrophages were closely associated with the proposed MSC niche. In all panels, the dashed white lines demarcate the proposed MSC niche. Bar = 50 μm

Having characterized the candidate niche-dwelling stem cells, we then tested whether previously identified candidate supportive cells are closely associated with the MSC niche and/or niche-dwelling MSCs. We examined the spatial relationship between specific candidate support cells with the proposed niche-dwelling stem cells (LRCs) at different stages of HO by co-staining LRC (CldU^+^) with different cell-specific markers, including endomucin/laminin (vasculature), SMI312 (neurites), and Typ (mast cells). CldU^+^ cells were closely associated with SMI312^+^ neurites (Fig. 4a), Typ^+^ mast cells (Fig. 4b), and endomucin/laminin^+^ vasculature (Fig. 4c, d). We also co-stained with macrophage markers including IBA1 in different stages of HO. Abundant IBA1^+^ macrophages were present in the lesion from the earliest stages (Fig. 4e), and more importantly, IBA1^+^ macrophages were closely associated with the proposed MSC niche from the condensation stage onward (Fig. 4f).

### BMP and Hh signaling co-regulate the MSC niche and the downstream events in a non-cell autonomous manner

**Fig. 5:**
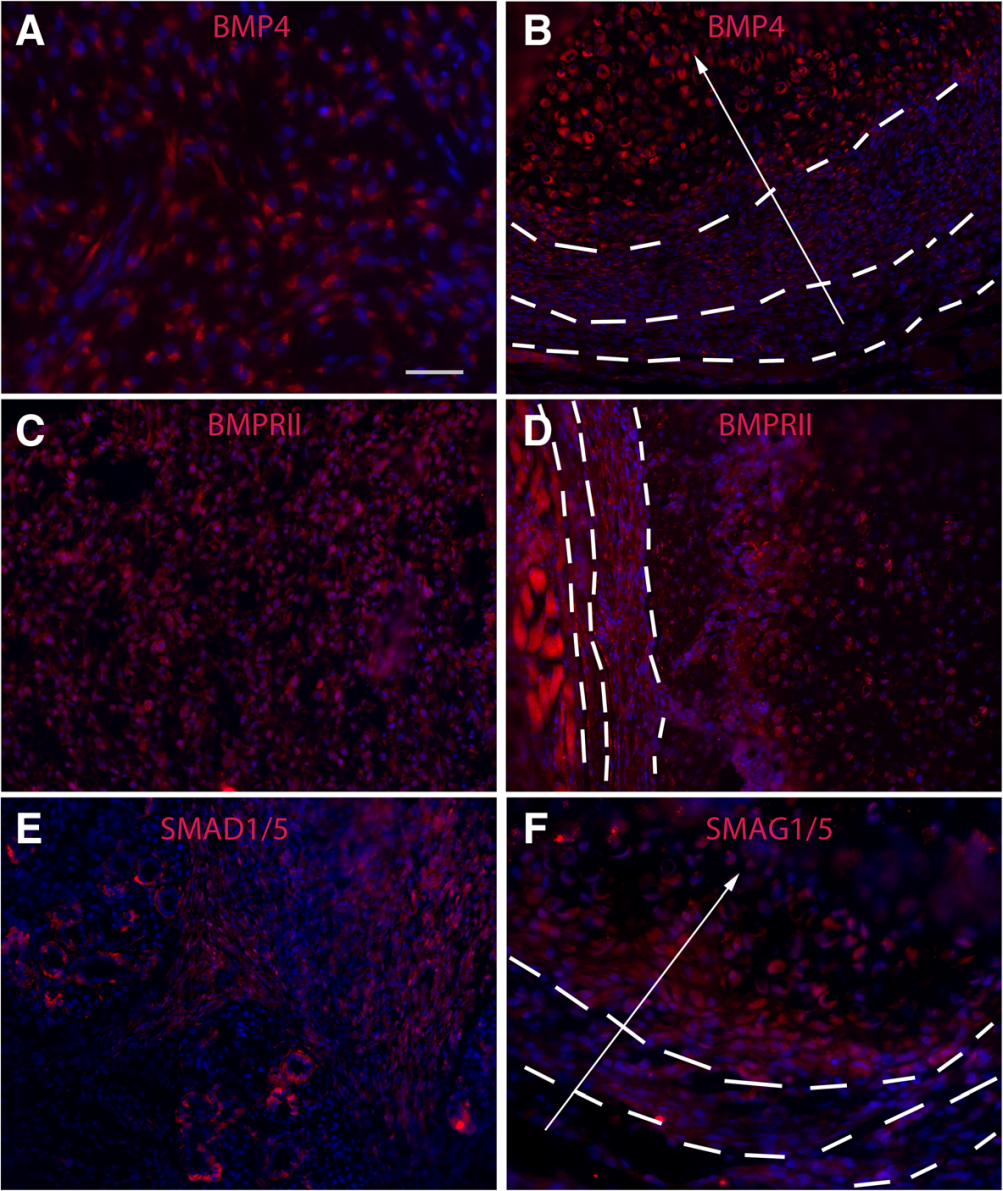
Characterization of BMP components in injury-induced lesions. **a**, **b** Typical image of BMP4 staining of early (**a**) and later (**b**) lesions. Note that BMP4 was upregulated but more or less evenly in the early lesion. However, a BMP4 gradient was apparent from the condensation stage onward, i.e., high BMP4 signal was located mostly in the inner core, and there was an obvious gradient (white arrow). **c**, **d** Typical image of BMPRII staining of early (**c**) and later (**d**) lesions. No obvious upregulation or gradient for BMPRII throughout the lesion. **e**, **f** Typical image of pSMAD1/5 staining of early (**e**) and later (**f**) lesions. Note that pSMAD1/5 showed a similar pattern as that of BMP4. Bar = 50 μm

To further understand the molecular mechanisms underlying HO, we first characterized the components of the BMP signaling pathway by IHC at different stages of HO. Our previous [6] and current studies indicated that the components of the BMP signaling pathway are upregulated dynamically during the HO process. For example, BMP4 is upregulated from the relatively early stage (around 3 days after injury), but the pattern of upregulation is dynamic, i.e., BMP signaling is upregulated first around the peripheral of the lesion (< 3 days after injury) [6] and then rather uniformly (3–6 days after injury) (Fig. 5a, e). From the condensation stage onward (> 6 days after injury), a stable BMP4 gradient was apparent with expression gradually increasing towards the inner core (Fig. 5b, f), echoing the observed LRC gradient. By contrast, there was no consistent increase or gradient of expression of the BMP receptors, BMPRII (Fig. 5c, d) or BMPRIa (data not shown) at any stage. The downstream signaling readout, pSMAD1/5, showed a pattern similar to that of BMP4 (Fig. 5e, f), suggesting that BMP signaling is both temporally and spatially regulated in the injury-induced lesion and that the stable BMP signaling gradient is likely responsible for the observed LRC gradient.

To further identify signaling pathways that facilitate the establishment/maintenance of the stable BMP signaling gradient, we examined several conserved signaling pathways in the lesions through IHC. But the tested patterns were either not consistent or did not correlate with the observed BMP signaling gradient in an obvious way. By contrast, in a previous study, we found that hedgehog (Hh) signaling components, such as Indian hedgehog (Ihh), Patched, and Gli1, were almost inversely correlated with the observed BMP signaling gradient [15], which suggested that Hh and BMP signaling might co-regulate the proposed MSC niche and the subsequent HO process, possibly through mutual inhibition. To test this idea and further understand the underlying mechanism, we reasoned that there might be at least three explanations for the inverse relationship between Hh and BMP: (a) Hh signaling could be an upstream regulator that inhibits BMP signaling. In this case, knockout of Hh signaling should release the inhibition and increase BMP signaling, which in turn would lead to enhanced HO, and (b) Hh signaling could be a downstream mediator that is inhibited by BMP signaling, but not vice versa. In this case, knockout of Hh signaling should not disturb BMP signaling directly but would inhibit the downstream process and cause decreased HO, or (c) Hh signaling is a downstream mediator of BMP signaling, and they mutually inhibit each other to a degree. In this case, knockout of Hh signaling should both disturb BMP signaling and inhibit the downstream process with decreased HO.

**Fig. 6:**
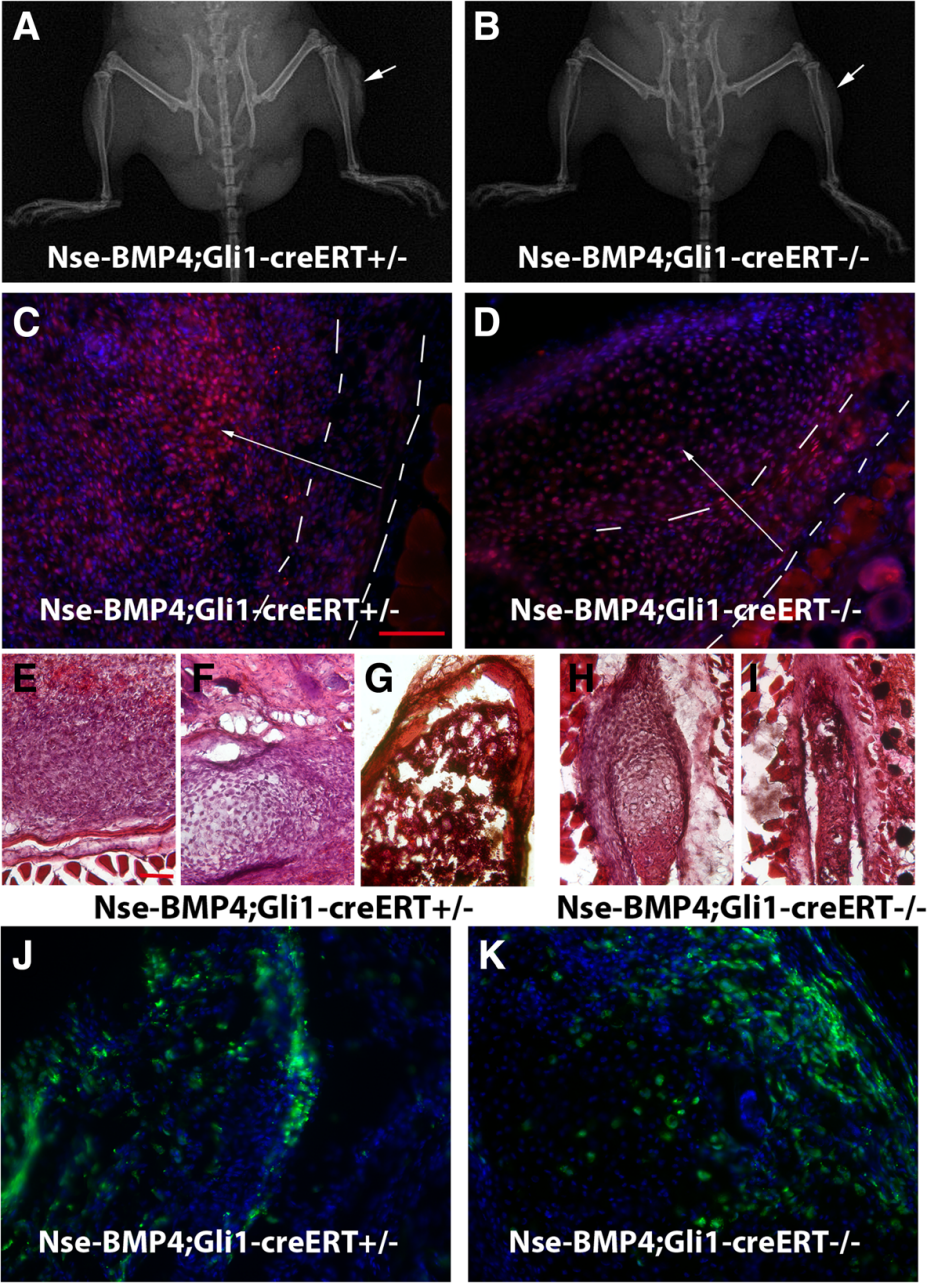
Functional consequences of conditional KO of Gli1 signaling. **a**, **b** Typical x-ray images of Nse-BMP4;Gli1-creERT^+/−^ (**a**) and Nse-BMP4;Gli1-creERT^−/−^ (**b**) mice after injury. Note that injury-induced HO was dramatically inhibited in Nse-BMP4;Gli1-creERT^−/−^ mice. **c**, **d** pSMAD1/5 staining of lesional tissues of Nse-BMP4;Gli1-creERT^+/−^ (**c**) and Nse-BMP4;Gli1-creERT^−/−^ mice (**d**). Note that overall BMP signaling was not obviously different between these two groups, but, unlike that of Nse-BMP4;Gli1-creERT^+/−^, in lesional tissues of BMP4;Gli1-creERT^−/−^ mice, the BMP signaling gradient was not observed. **e**–**i** H&E staining showed that, in BMP4;Gli1-creERT^+/−^ mice (**e–g**), all stages of the typical HO process, i.e., early fibroproliferative (**e**), chondrogenesis (**f**), and mature HO (**g**), were robustly observable. However, in Nse-BMP4;Gli1-creERT^−/−^ mice, even though the whole HO process was dramatically inhibited, not all stages were equally affected (**h**, **i**). Note that chondrogenesis was less affected, while the fibroproliferative region was severely underdeveloped and could only be found around the cartilage core, and the most mature structure found in Nse-BMP4;Gli1-creERT^−/−^ mice was underdeveloped HO without well-developed trabeculae or bone marrow (**i**). Intriguingly, based on morphology, at least in the chondrogenesis stage, the MSC niche was still observable (only mildly affected). **j**, **k** In comparison to Nse-BMP4;Gli1-creERT^+/−^; Zsgreen (**j**), pre-existing Gli1-creERT labeled stem/progenitor cells in Nse-BMP4;Gli1-creERT^−/−^; Zsgreen mice could survive and contribute to the MSC niche in the absence of ongoing Gli1 signaling (**k**). **c**, **d**, **j**, and **k** are on the same scale, bar = 100 μm; **f**–**i** are on the same scale, bar = 100 μm

To test this hypothesis, we first generated conditional Gli1 null mice with high BMP signaling (i.e., Gli1^−/−^;Nse-BMP4) and then compared the injury-induced HO in these two groups(i.e., with or without Gli1 signaling) after TAM induction in adult mice. We found that HO was dramatically inhibited in Nse-BMP4;Gli1-creERT^−/−^ mice (Fig. 6a, b), essentially excluding the first possibility (i.e., Hh is not a upstream inhibitor). To further distinguish between the other two possibilities, we directly evaluated pSMAD1/5 levels in these two groups and found that the overall pSMAD1/5 levels were comparable between two groups (Fig. 6c, d); however, the typical BMP signaling gradient was not observed in the lesions of Nse-BMP4;Gli1-creERT^−/−^ mice from the condensation stage onward (Fig. 6d). This strongly supports the third possibility, i.e., Hh and BMP mutually inhibit each other. More detailed histological study (Fig. 6e–i) found that although the HO process was dramatically inhibited in Nse-BMP4;Gli1-creERT^−/−^ mice (Fig. 6h, i), not all stages were equally affected (comparing Fig. 6e–g and h, i), i.e., chondrogenesis was only mildly affected (Fig. 6d, h), further suggesting the involvement of the potential unknown stage-specific factor(s).

To further clarify whether the effect of loss-of-function of Hh signaling is cell autonomous, we generated triple mutant Nse-BMP4;Gli1-creERT^−/−^;Zsgreen mice by crossing Nse-BMP4;Gli1-creERT^+/−^ and Gli1-creERT^+/−^;Zsgreen. The underlying rationale was that if the role of Hh signaling were cell autonomous, pre-existing Gli1^+^ stem/progenitor cells would likely not participate in establishing the injury-induced MSC niche and the downstream HO process without ongoing Hh signaling. To better understand Fig. 6, we need to clarify the nomenclature used for the animal models: (1) the Gli1-CreERT allele was created by knocking-in CreERT into the endogenous Gli allele, which therefore conditional expresses CreERT instead of endogenous Gli1. Thus, the Gli1-CreERT allele represents a loss-of-function allele of Gli1. (2) For this reason, we can treat the homozygous mice Gli1-CreERT^−/−^ essentially as Gli1^−/−^ mice (i.e., Gli1-CreERT^−/−^≈Gli1^−/−^), while heterozygous Gli1-CreERT^+/−^ mice have a reduced copy number of Gli1. (3) Thus, the phenotypic change was primarily due to the change in the copy number of Gli1 (i.e., Gli signaling) (Additional file 11: Figure S8), although it is theoretically possible that the phenotypic change could be attributed to a copy number change of CreERT, which has been changed concurrently.

We next directly tested whether pre-existing Gli1^+^ stem/progenitor cells in Nse-BMP4;Gli1-creERT^−/−^;Zsgreen mice could survive and contribute to the MSC niche and the subsequent HO process in the absence of ongoing Gli1 signaling. We found that even in the absence of ongoing Gli1 signaling, pre-existing Gli1^+^ stem/progenitor cells (GFP^+^) could still contribute to proposed MSC niche and subsequent HO lesions (Fig. 6j, k), although the overall HO process was significantly inhibited. This suggests that Hh signaling is at least partially non-cell autonomous.

In addition, we also explored other potential niche supportive molecules, including key extracellular matrix proteins, such as collagen IV (Col4), collagen VI (Col6), tenascin C (TEN), laminin (LAM), and chondroitin sulfate proteoglycan (CSPG). Overall, the tested extracellular matrix proteins were all increased in HO lesions, but with different patterns (Additional file 12: Figure S9). Specifically, LAM and Col4 were mainly involved in forming micro-tubular (vasculature) structures in the proposed MSC niche, which may reflect de novo angiogenesis. Col6, a form of collagen primarily associated with the extracellular matrix of skeletal muscle, was more specifically upregulated in the proposed MSC niche. In early lesions, TEN was diffusedly upregulated, but in later stages, TEN was enriched primarily in the transient and mature domains, but not the MSC niche.

Overall, our current data support the following working model: unlike normal skeletogenesis, the potential core process is the formation of a relatively stable injury-induced local microenvironment (MSC niche), which is composed of niche-dwelling progenitor/stem cells (including at least Tie2-cre-, Gli1-creER-, and Glast-creERT-labeled cells) and niche supportive cells (including mast cells, neurites, vasculature, and macrophages). The formation of the MSC niche likely initiates the pathological osteogenic cascade, under the co-regulation of BMP and Hh signaling through feedback and non-cell autonomous mechanisms (Additional file 13: Figure S10).

## Discussion

Our current understanding of the pathophysiology of HO is limited, and there has been no single hypothesis that could explain all experimental and clinical observations. In this study, we proposed a hypothesis and sought to explore experimentally a potential common core mechanism that could explain most research and clinical observations. We believe that the emergence of a good working model will hopefully facilitate development of more effective therapeutic interventions.

The seminal finding of this study was the identification of the unique LRC pattern (zonal pattern or domains) in injury-induced lesions from the condensation stage onward. More importantly, this three-domain/zone pattern is potentially a common underlying mechanism, since it is not only observed in our animal model of injury-induced, BMP-dependent HO, but also in pathological specimens from aHO and FOP patients. This stereotyped pattern is strongly reminiscent of some endogenous stem cell niches, such as the neural stem cell (NSC) niche in the adult brain [27], which features well organized micro-domains, i.e., the ventricular zone (VZ), subventricular zone (SVZ), and intermediate zone (IZ).

Previous lineage tracing demonstrated that many mesenchymal stem/progenitor subpopulations, including Prx-cre [28], Scleraxis-cre [29], Nfatc1-cre [30], MX1-cre [31], and Wnt1-cre [32], in addition to the three subpopulations (Tie2-cre, Gli1-creER, and Glast-creERT) we have tested in this study, contribute significantly to HO [13–15, 31]. A key question in this study was whether these subpopulations contribute to HO through the MSC niche. For practical reasons, we focused on three Cre-labeled populations and, based on our clonal analysis and loss-of-function study, concluded that the Tie2-cre-, Gli1-creER-, and Glast-creERT-labeled stem/progenitor subpopulations behave like niche-dwelling stem cells.

The findings using DTA-induced depletion further strengthened the causal relationship, but also raised an interesting question: why did the same Cre lines generate such dramatic phenotypes when combined with ROSA26-eGFP-DTA allele, while in the clonal analysis the labeling efficiency was apparently low? Even though the exact reason is still unclear, at least two explanations can be proposed: (1) in the clonal analysis (with R26R-Confetti reporter line), not all recombination events can be detected unequivocally with comparable efficiency, which leads to inaccurate estimation of recombination efficiency; in other words, the real recombination efficiency could be much higher with Gli1-creER or Glast-creERT; or (2) less than optimal efficiency of depletion in the early stage could still cause dramatic downstream phenotypes, simply because of the clonal nature of the niche-dwelling stem cells; stated otherwise, depletion of a few early stem cells could potentially cause severe downstream phenotype.

Previous studies indicated that many different types of cells, including macrophages [20], nerve fibers [7], mast cells [8, 9], and vasculature (endothelial cells) [33], are involved in the HO process. Our current study further indicates that many of these cells are closely and persistently associated with the MSC niche, suggesting that these cells might be niche supportive cells. However, this study did not exclude the possibility that lymphocytes or other immune cells still play important roles in regulating the abnormal injury/immune response, a pre-condition for MSC niche development. The critical question is how the early pre-conditions evolved into relatively stable and zonal structure, which initiates the definite HO process, rather than undergoing the normal wound healing process? The study of BMP and Hh signaling and the possible co-regulation provided a tentative mechanism, i.e., the self-organization/assembly of the MSC niche under the co-regulation of BMP and Hh signaling in response to the early pre-conditions, could ultimately tip the balance of homeostasis (normal healing) towards the formation of an MSC niche and HO.

Numerous studies have implicated various signaling pathways, such as BMP, wnt/β-cat, FGF, Hh signaling, and probably other conserved pathways [17], in the HO process. But the exact underlying mechanisms of molecular signaling and cross-talk among these pathways are likely complex and context-dependent [15]. In this regard, it is worthy to stress that mutually inhibition of Hh and BMP might not be the only mechanism in this context, and other currently unknown mechanisms must also be involved, since if this were the only mechanism, Gli1^−/−^ should not only dysregulate the gradient but also lead to significant upregulation of overall BMP signaling. But this phenomenon has not been observed in the lesion.

Notably, preexisting Gli1^+^ progenitors/stem cells still contributed to the MSC niche without ongoing Gli1 signaling, even though the overall HO process was inhibited in this context. This suggests non-cell autonomous effects of Hh signaling. The role of Gli1 signaling in HO was drawn from two different loss of function animal models (i.e., Nse-BMP4;Gli1-creERT^−/−^;Zsgreen and Nse-BMP4;Gli1-creERT;ROSA26-eGFP-DTA mice) and is also consistent with the findings in a prior loss of function animal study that used an Hh inhibitor [15]. Previous studies also indicated that Hh-Gli1 itself can induce early osteoblast differentiation, that there is a redundant role for Gli2, and that Hh is involved in the repressor function of Gli3 in osteogenesis [34]. Other studies suggest that Hh signaling also may play an important role in postnatal osteogenesis in the setting of stress fracture healing [35]. More interestingly, in a pathological context, Regard et al. also demonstrated that elevated Hh signaling might be the key underlying mechanism of progressive osseous heteroplasia (POH), a type of rare intramembranous HO.

Our current study also may have broader implications for development of translational applications of MSC. Although MSC possess many desirable features for regenerative medicine, therapeutic applications of MSC have been slow to emerge. One major challenge is that we know relatively little about the behavior of MSC in vivo and about how appropriate niches are formed that facilitate differentiation of the cells into the desired phenotypes. In part, this reflects the lack of a practical tool, and in particular an animal model, for studying the mechanisms that promote niche formation and programmed lineage commitment. In this regard, our working model may be helpful for the understanding of such mechanisms in vivo that will be crucial for development of MSC-related cell therapies.

The main focus of this study was to identify and characterize the injury-induced MSC niche. Injury-induced microenvironments have been demonstrated in several injury models, especially in the CNS [36–38]. But the basic structures of the niche, such as niche-dwelling stem cells and niche supportive cells and molecules, have not been systematically studied in those reports; therefore, the underlying mechanisms that regulate this type of injury-induced microenvironment have been largely unknown. In fact, even for the well-established, endogenous hematopoietic stem cell niche [39, 40], the structure and functional details of the niche are still unclear or controversial, more than 40 years after its original discovery [41]. Since this is only the a proof-of-concept study that tries to characterize an injury-induced stem cell niche outside of the nervous system, many important aspects have not been covered.

There are also several caveats regarding our findings: (1) Since the central hypothesis of this study aims to provide a potential common core mechanism for HO, testing this concept in multiple different models will be necessary to validate the extent to which it is generalizable. Even though we have tested this idea in injury-induced, BMP-dependent animal model (Nse-BMP4) and in human aHO and FOP samples, more detailed studies in different models, such as ACVR1^[^^R206H]^ model,   are warranted. (2) Although the LRC approach is widely used to label and identify potential stem cell populations in many different tissues, it has a series of limitations [42]. (3) Due to our failure to construct the relevant transgenic mice, we were unable to fully characterize the effects of loss of function of the Tie2-cre-labeled subpopulation. (4) Other abovementioned HO contributing subpopulations, such as MX1-cre and SCX-cre [31], have not been tested. (5) Conditionally depleting the Glast-creERT or Gli1-creERT subpopulations produced similar phenotypes, which strengthened the central hypothesis, but we have not delineated the exact relationship between these two candidate populations or their hierarchical relationship with other candidate populations. (6) The precise constellation of factors that regulate BMP signaling and the observed BMP signaling gradient are still largely unknown. Despite these limitations, our proof-of-concept study, for the first time, provides evidence of a potential core mechanism for HO.

## Conclusions

Available data supported the central hypothesis, i.e., the formation of a relatively stable injury-induced local microenvironment (niche) might be a crucial core process, and this niche regulates the proliferation and osteogenic differentiation of mesenchymal stem cells (MSCs). The important implication  for HO treatment is that therapeutic approaches must consider several different disease-specific factors as parts of the functional unit, instead of treating one factor at a time. The insights gained from this study have broad implications not only for the prevention and treatment of HO, but also for the potential translational applications of MSCs in a variety of injury or regenerative-related disorders.

## Additional files

Additional file 1: **Table S1.** Summary of clinical data of HO patients. (DOCX 15 kb)

Additional file 2: **Table S2.** Summary of the histomorphometric analysis of 8 aHO samples from 8 patients. (DOCX 75 kb)

Additional file 3: **Figure S1.** Representative X-ray/CT images of 8 patients with aHO. The number in each panel is the patient’s coded serial number after deidentification of the images. (JPG 603 kb)

Additional file 4: **Figure S2.** Representative H&E images of 8 patients with aHO. The number in each panel is the patient’s coded serial number after deidentification of the tissues. (JPG 2131 kb)

Additional file 5: **Figure S3.** Validation of the dual-pulse CldU and IdU labeling procedure. To validate this procedure, we used two positive controls (A&B), and two negative controls (C&D). As expected, we observed plentiful specific staining, including CldU^+^/IdU^−^ (red arrows), CldU^+^/IdU^+^ (yellow arrows) and CldU^−^/IdU+ (green arrows) cells in intestinal mucosa (A) & hair follicles (B) but not in muscle (C&D). E) Experimental paradigm for dual-pulse labeling procedure. A-D are on the same scale, Bar = 50 μm. (TIF 9688 kb)

Additional file 6: **Figure S4.** LRC cells co-labeled with typical MSC markers. In the early stages of the lesion (A&C), and proposed niche (B&D), many CldU^+^/IdU^−^ (quiescent stem cells) co-labeled with Stro1(A&B) and S100A4 (C&D). A-D are on the same scale, Bar = 50 μm. (TIF 9632 kb)

Additional file 7: **Figure S5.** The distribution of Cre-labeled cells outside of the target regions. A&B) the distribution of Gli1-creERT-labeled cells in Nse-BMP4;Gli1-creERT;R26R-Confetti mice outside of the target regions, i.e., A) in normal skeletal bone (growth plate of femur), and B) in differentiated core of chondrocyte of HO, away from the newly formed zonal region. C&D) the distribution of Glast-creERT labeled cells in Nse-BMP4;Glast-creERT;R26R-Confetti mice outside of the target regions, i.e., C) in the cerebellum, consistent with the known expression pattern in Bergmann glia, and D) in the skeletal muscle interstitium. E&F) the distribution of Tie2-cre labeled cells in Nse-BMP4;Tie2;R26R-Confetti and Nse-BMP4;Tie2-cre;Zsgreen mice outside of the target regions, i.e., E) The pattern of labeled cells in the adult brain of Nse-BMP4;Tie2-cre;Zsgreen, consistent with the known vascular expression pattern. F) The pattern of labeled cells in the early lesion of Nse-BMP4;Tie2-cre;R26R-Confetti. Note that the morphology of some labeled cells is consistent with the known vascular pattern. A-F are on the same scale, Bar = 50 μm. (TIF 11999 kb)

Additional file 8: **Table S3.** Summary of the histomorphometric analysis of Nse-BMP4;Glast-creERT;ROSA26-eGFP-DTA mice with or without TAM treatment. (DOCX 72 kb)

Additional file 9: **Figure S6.** Conditional depletion of Glast-creERT+ cells resulted in less severe yet “typical” HO. A&B) Gross image of HO harvested from TAM treated (A) and control (B) Nse-BMP4;Glast-creERT;ROSA26-eGFP-DTA mice after injury. Note that the gross morphology of HO in both groups was similar but the HO in the TAM treated group was smaller. Also note that a significant portion of harvest HO was not mature (without red bone marrow), which argued that quantification the immature HO with micro-CT could be misleading. C-H) Typical H&E images from treated (C, E &G) and control (D, F&H) groups both demonstrate typical features of fibro-proliferative (C&D), chondrocyte (E&F) and mature HO (G&H), though subtle differences do exist between the two groups. C-H are on the same scale, Bar = 50 μm. (TIF 18128 kb)

Additional file 10: **Figure S7.** Gli1-creERT-mediated DTA expression inhibited injury-induced HO. A&B) Typical x-ray images of control (A) and TAM treated (B) Nse-BMP4;Gli-creERT;ROSA26-eGFP-DTA mice after injury. C) HO incidence in control and TAM treated group. D) Quantification of wet weight of HO in the control and TAM treated groups. Note that depletion of Gli1-creERT-labeled cells partially inhibited but did not completely block HO. E) Typical fluorescence images from TAM treated (E) and control (F) Nse-BMP4;Gli1-creERT;ROSA26-eGFP-DTA mice. Note that in the TAM treated group (E), GFP- (recombined) cells were rarely found. G&H) H&E staining of sections from TAM treated (G) and control (H) Nse-BMP4;Gli1-creERT;ROSA26-eGFP-DTA mice. Note that both fluorescence images and H&E staining suggest that the proposed MSC domain (within dashed lines) was thinner in the TAM treated group. E-H are on the same scale, Bar = 50 μm. (TIF 15685 kb)

Additional file 11: **Figure S8.** Evidence of depletion of Gli1 in the target cells. The depletion of Gli1 in the target cells was confirmed by staining the tissue sections of Nse-BMP4;Gli1-creERT^−/−^;Zsgreen mice (A, low power &B, high power), and the tissues of Nse-BMP4;Gli1-creERT^+/−^;Zsgreen (C, low power &D, high power) mice with Gli1 antibody. Note that there is no specific staining of Gli1 (red) in the lesional tissues from Nse-BMP4;Gli1-creERT^−/−^;Zsgreen mice, while the specific staining of Gli1 (red) was observed in the Zsgreen+ cells in the proposed MSC niche in lesional tissues from Nse-BMP4;Gli1-creERT^+/−^;Zsgreen mice. (JPG 822 kb)

Additional file 12: **Figure S9.** Characterization of candidate niche supportive ECM molecules. A) Col4 was mainly involved in forming microtubular structures in the proposed MSC niche. B) Col6 was more ubiquitously upregulated in the proposed MSC niche. C) Interestingly, Col6 was closely associated with Tenascin C (TEN) only in early lesions. D) TEN was diffusedly upregulated in the early stages, but in the later stages, TEN was enriched mostly in mature domains. E&F) Similar to TEN, Chondroitin sulfate proteoglycan (CSPG) was also more or less evenly upregulated in early lesions but it became more defined as the proposed MSC niche formed. In contrast, laminin (LAM) was mainly involved in forming micro-tubular structures in the proposed MSC niche. A-F are on the same scale, Bar = 50 μm. (TIF 9818 kb)

Additional file 13: **Figure S10.** Working model of injury-induced MSC niche. Injury-induced local microenvironment (MSC niche) is composed of niche-dwelling progenitor/stem cells (including at least Tie2-cre, Gli1-creER and Glast-creERT labeled cells) and niche supportive cells (including mast cells, neurites, vasculature and macrophages). The formation of the MSC niche likely initiates the pathological osteogenic cascade, under the co-regulation of BMP and Hh signaling through feedback and non-cell autonomous mechanisms. (TIF 1825 kb)

## Abbreviations

aHO: Acquired heterotopic ossification
AIR: Abnormal injury/inflammatory responses
BMPs: Bone morphogenetic proteins
DTA: Diphtheria toxin A
FOP: Fibrodysplasia ossificans progressiva
Hh: Hedgehog
HO: Heterotopic ossification
LRC: Label-retaining cell
MSC: Mesenchymal stem cell
TAM: Tamoxifen
