# Supplementary material for: BMP-dependent, injury-induced stem cell niche as a mechanism of heterotopic ossification
Source: Stem Cell Res Ther. 2019 Jan 11;10:14. doi: 10.1186/s13287-018-1107-7 (PMC6329163; doi:10.1186/s13287-018-1107-7)
Supplement: Supplementary file 1 — Table S1. Summary of clinical data of HO patients. (DOCX 15 kb) [file 13287_2018_1107_MOESM1_ESM.docx]

Additional file 1

Table S1 Summary of clinical data of HO patients

| **Sample No.** | **Sex** | **Age**  **(years)** | **Brief disease history, diagnosis & treatment** | **Target tissues?** | **Zonal pattern?** |
| --- | --- | --- | --- | --- | --- |
| 2211874/460012 | male | 56 | Car accident caused left femoral head necrosis, and left hip HO. Received total hip arthroplasty and HO resection. | yes | yes |
| 730205/374863 | female | 46 | Motorcycle accident caused left hip HO. Received HO resection. | yes | yes |
| 2299638/421033 | male | 41 | Car accident caused multiple fractures. Received internal fixation and developed left hip HO subsequently, which was resected. | yes | yes |
| 2030139/374503 | male | 37 | Running accident caused Left elbow (ulnar coronoid) fracture. Received internal fixation, and developed HO subsequently, which was resected. | yes | yes |
| 2205342/403474 | female | 35 | Bicycle accident caused left elbow HO. Received HO resection. | yes | yes |
| 2459198/396954 | male | 39 | Basketball accident caused right ulnar fracture. Received internal fixation, and developed HO subsequently, which was resected. | yes | yes |
| 280954/522530 | female | 29 | 16 months after operation of the left acetabular fracture, internal fixation failed. Developed femoral head necrosis with HO. Received total hip replacement and HO resection. | No? | No? |
| 2019855/371289 | male | 9 | Bicycle accident caused right ulnar coronoid fracture. Received internal fixation, and developed HO subsequently, which was resected. | No? | No? |
| Code109 | female | 7 | Wide spread unknown swelling with restricted joint motion of the upper arm. Received biopsy before the final diagnosis of Fibrodysplasia ossificans progressiva | Yes | yes |
| Code21 | male | 4 | Unidentified subcutaneous nodule. Received biopsy before the final diagnosis of Fibrodysplasia ossificans progressiva | no | no |
| Code29 | male | 15 | Unidentified subcutaneous nodule. Received biopsy before the final diagnosis of Fibrodysplasia ossificans progressiva | yes | yes |
