# Supplementary material for: BMP-dependent, injury-induced stem cell niche as a mechanism of heterotopic ossification
Source: Stem Cell Res Ther. 2019 Jan 11;10:14. doi: 10.1186/s13287-018-1107-7 (PMC6329163; doi:10.1186/s13287-018-1107-7)

Additional file 2

**Table S2 Summary of the histomorphometric analysis of 8 aHO samples from 8 patients**


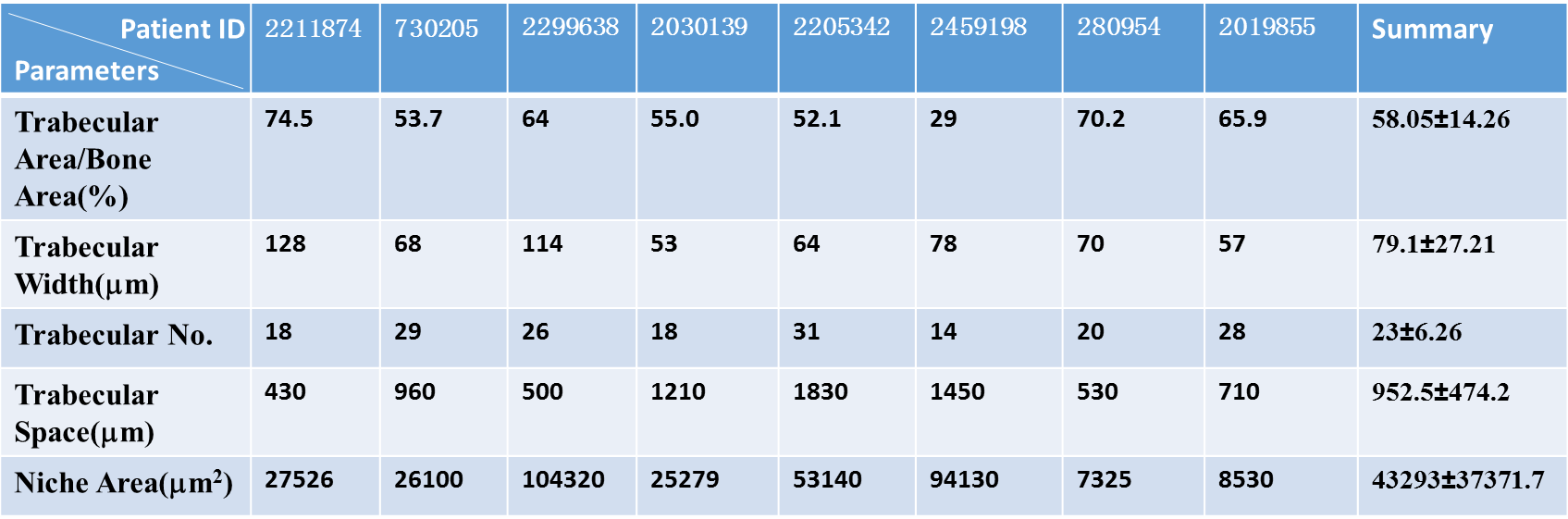

Supplement: Supplementary file 2 — Table S2. Summary of the histomorphometric analysis of 8 aHO samples from 8 patients. (DOCX 75 kb) [file 13287_2018_1107_MOESM2_ESM.docx]
