# Supplementary figures and images for: BMP-dependent, injury-induced stem cell niche as a mechanism of heterotopic ossification
Source: Stem Cell Res Ther. 2019 Jan 11;10:14. doi: 10.1186/s13287-018-1107-7 (PMC6329163; doi:10.1186/s13287-018-1107-7)

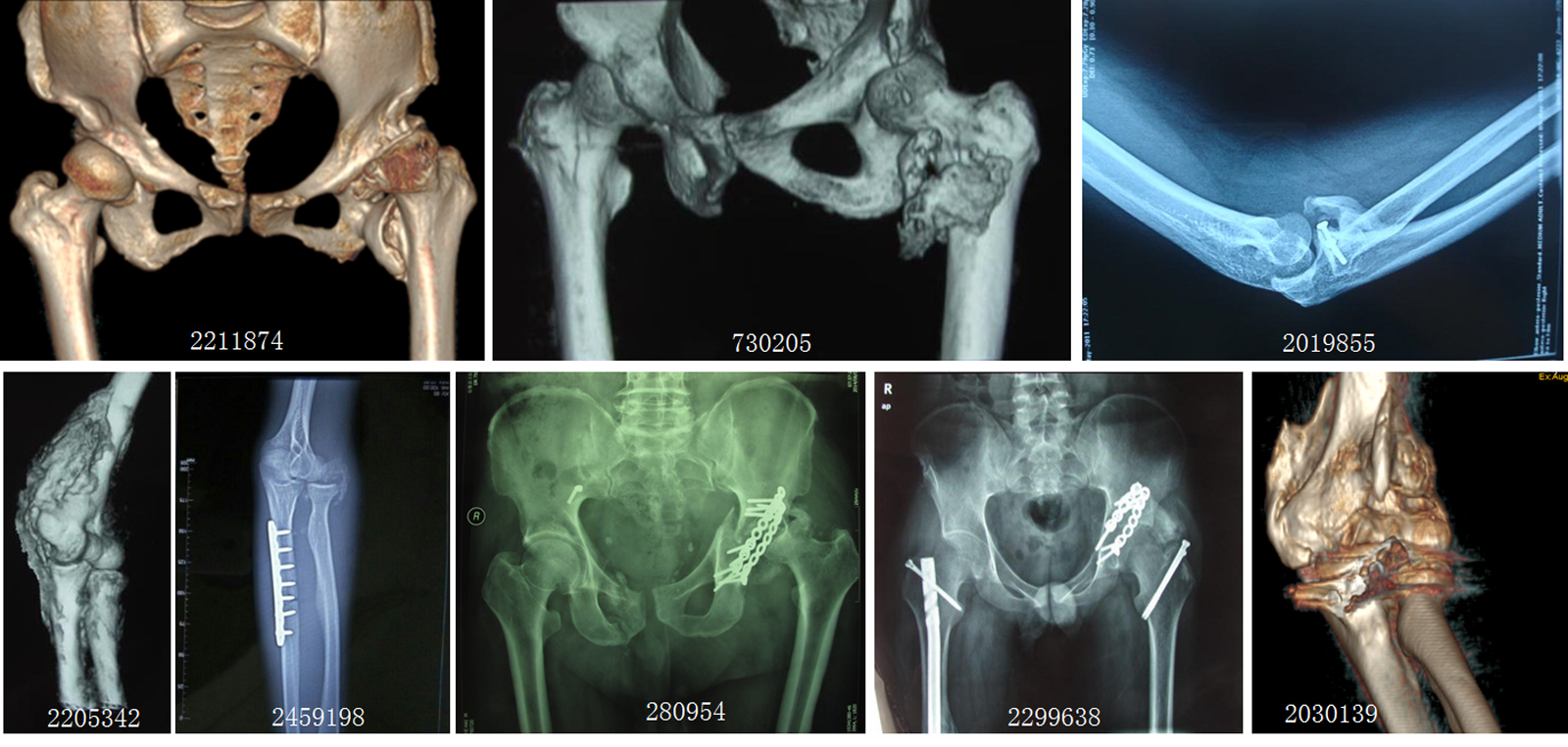

Supplement: Supplementary file 3 — Figure S1. Representative X-ray/CT images of 8 patients with aHO. The number in each panel is the patient’s coded serial number after deidentification of the images. (JPG 603 kb) [file 13287_2018_1107_MOESM3_ESM.jpg]

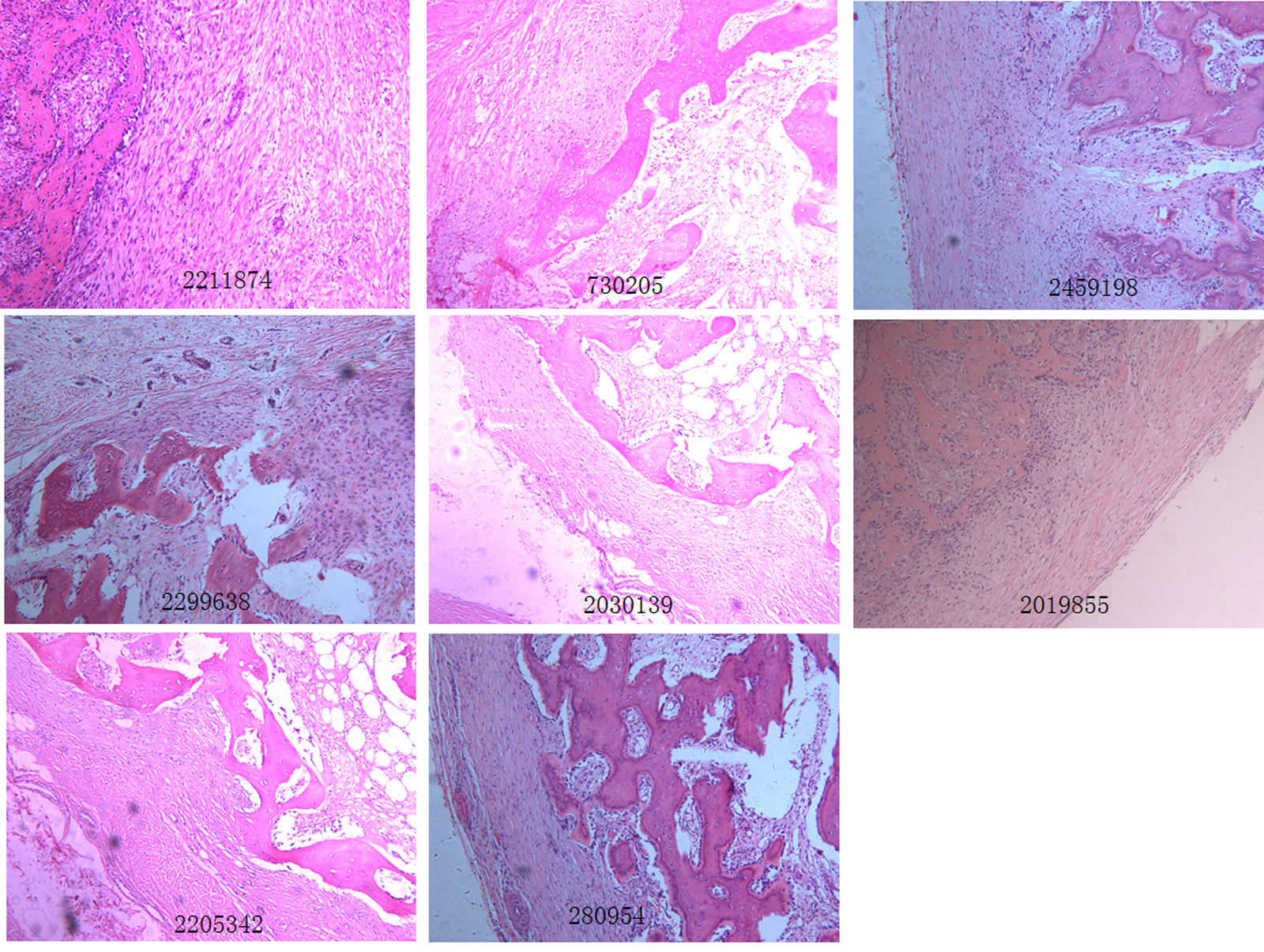

Supplement: Supplementary file 4 — Figure S2. Representative H&E images of 8 patients with aHO. The number in each panel is the patient’s coded serial number after deidentification of the tissues. (JPG 2131 kb) [file 13287_2018_1107_MOESM4_ESM.jpg]

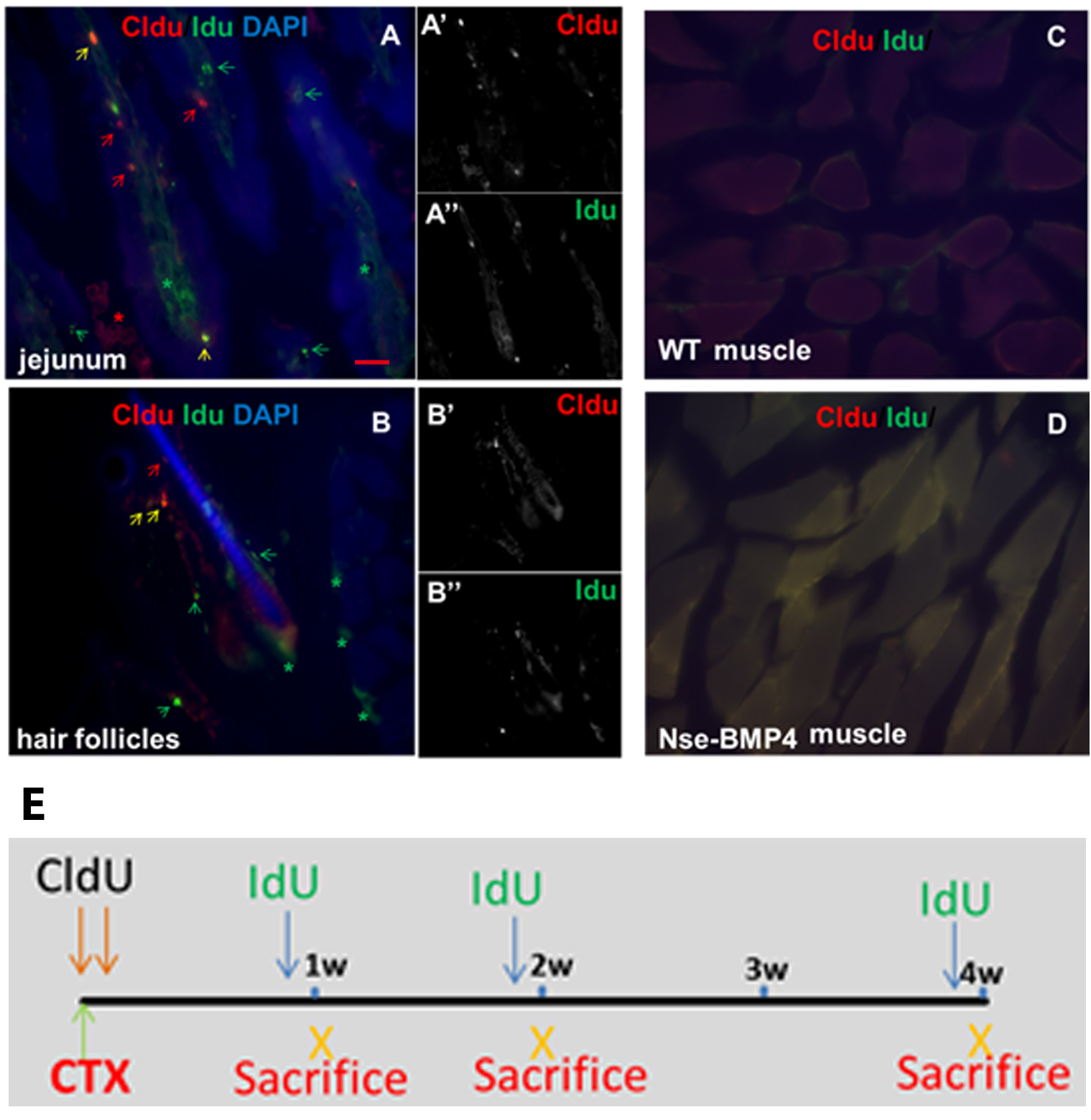

Supplement: Supplementary file 5 — Figure S3. Validation of the dual-pulse CldU and IdU labeling procedure. To validate this procedure, we used two positive controls (A&B), and two negative controls (C&D). As expected, we observed plentiful specific staining, including CldU+/IdU− (red arrows), CldU+/IdU+ (yellow arrows) and CldU−/IdU+ (green arrows) cells in intestinal mucosa (A) & hair follicles (B) but not in muscle (C&D). E) Experimental paradigm for dual-pulse labeling procedure. A-D are on the same scale, Bar = 50 μm. (TIF 9688 kb) [file 13287_2018_1107_MOESM5_ESM.tif]

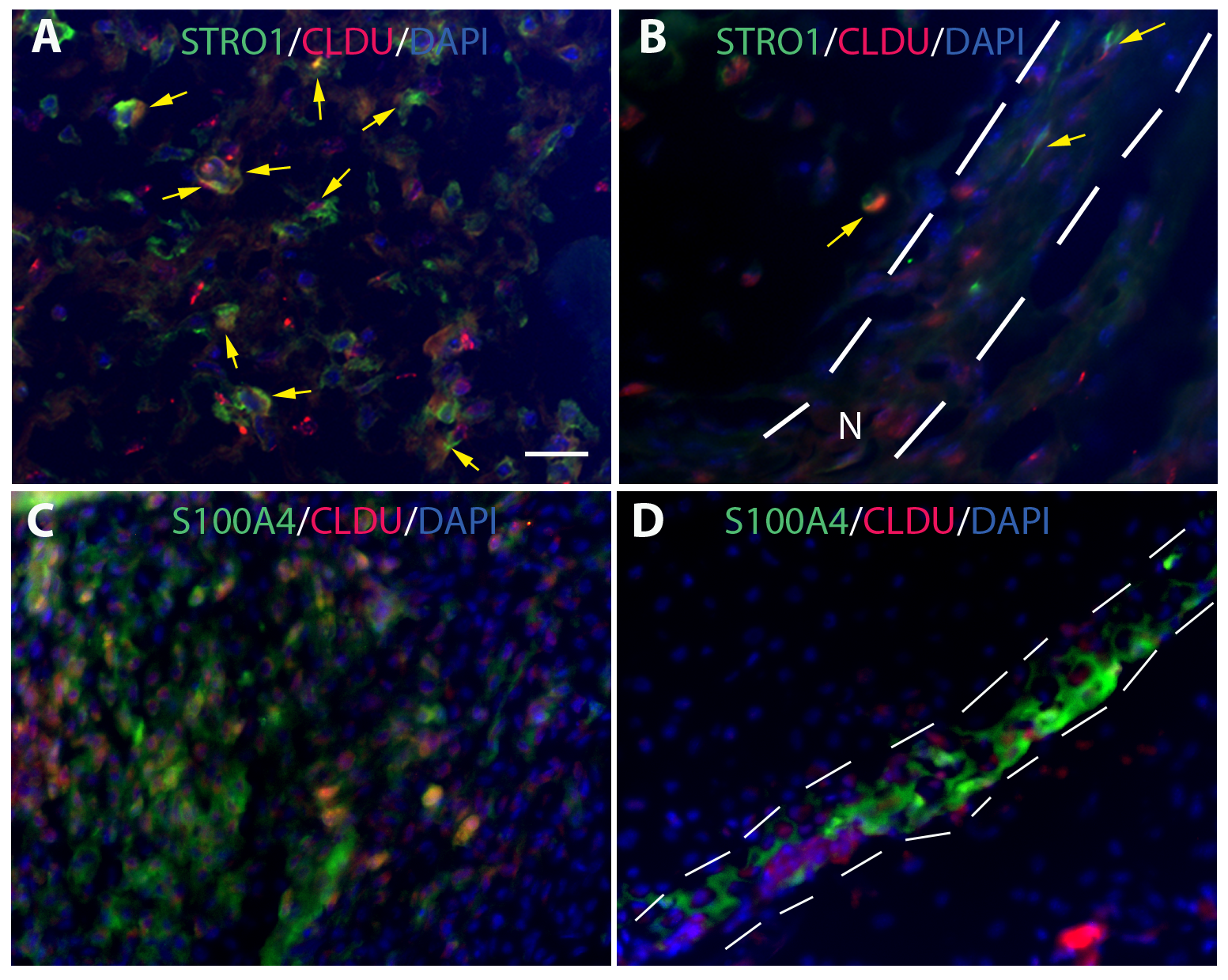

Supplement: Supplementary file 6 — Figure S4. LRC cells co-labeled with typical MSC markers. In the early stages of the lesion (A&C), and proposed niche (B&D), many CldU+/IdU− (quiescent stem cells) co-labeled with Stro1(A&B) and S100A4 (C&D). A-D are on the same scale, Bar = 50 μm. (TIF 9632 kb) [file 13287_2018_1107_MOESM6_ESM.tif]

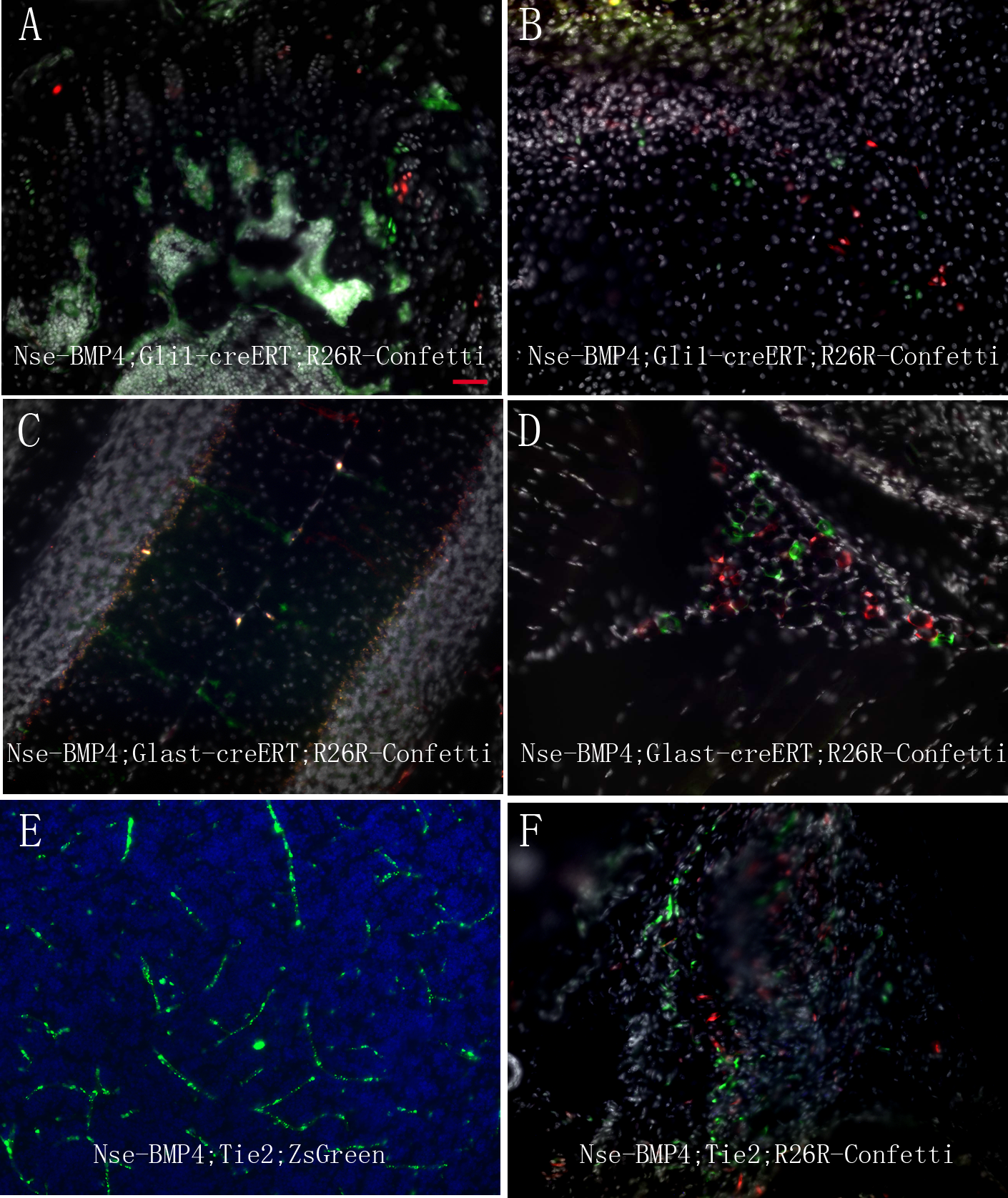

Supplement: Supplementary file 7 — Figure S5. The distribution of Cre-labeled cells outside of the target regions. A&B) the distribution of Gli1-creERT-labeled cells in Nse-BMP4;Gli1-creERT;R26R-Confetti mice outside of the target regions, i.e., A) in normal skeletal bone (growth plate of femur), and B) in differentiated core of chondrocyte of HO, away from the newly formed zonal region. C&D) the distribution of Glast-creERT labeled cells in Nse-BMP4;Glast-creERT;R26R-Confetti mice outside of the target regions, i.e., C) in the cerebellum, consistent with the known expression pattern in Bergmann glia, and D) in the skeletal muscle interstitium. E&F) the distribution of Tie2-cre labeled cells in Nse-BMP4;Tie2;R26R-Confetti and Nse-BMP4;Tie2-cre;Zsgreen mice outside of the target regions, i.e., E) The pattern of labeled cells in the adult brain of Nse-BMP4;Tie2-cre;Zsgreen, consistent with the known vascular expression pattern. F) The pattern of labeled cells in the early lesion of Nse-BMP4;Tie2-cre;R26R-Confetti. Note that the morphology of some labeled cells is consistent with the known vascular pattern. A-F are on the same scale, Bar = 50 μm. (TIF 11999 kb) [file 13287_2018_1107_MOESM7_ESM.tif]

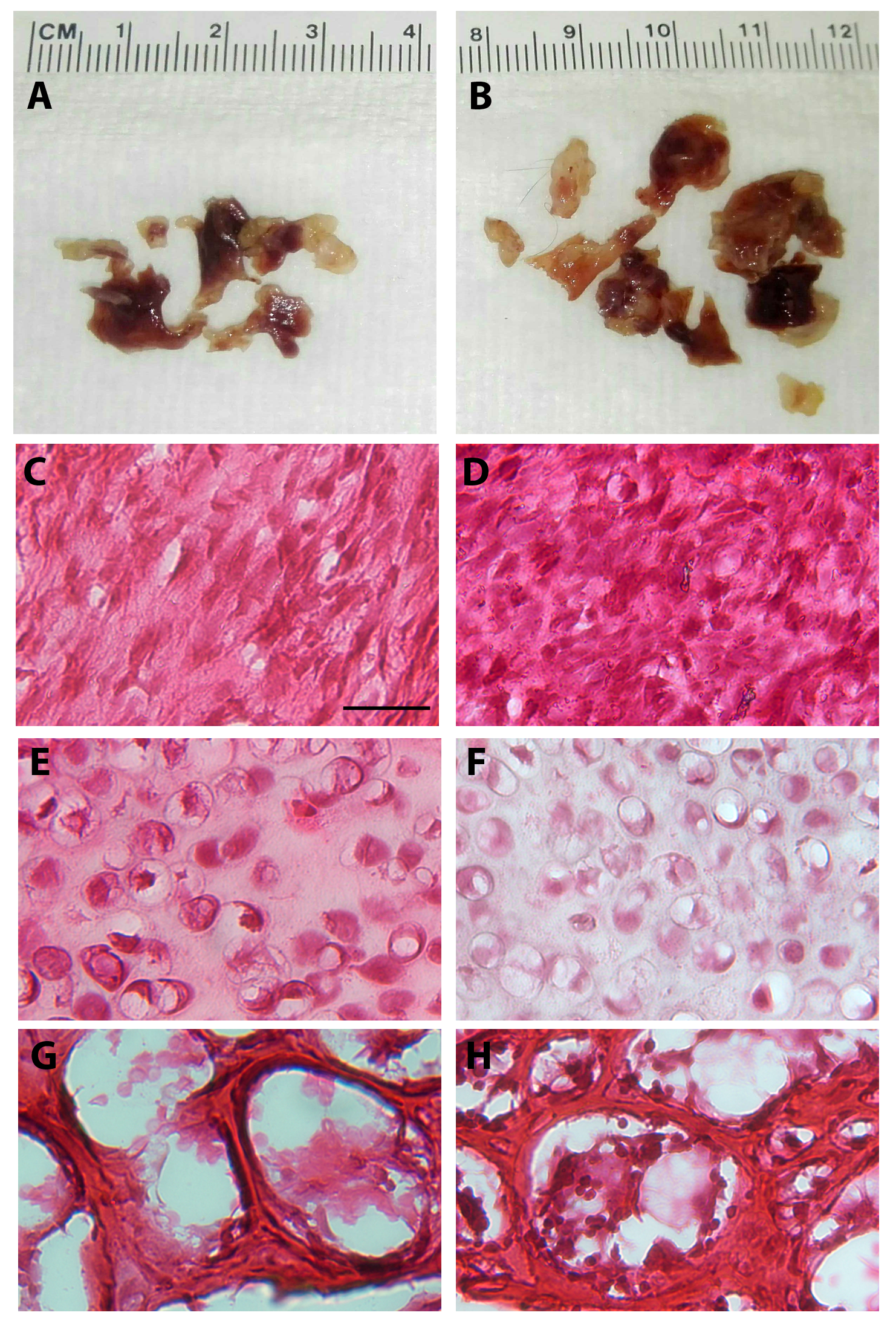

Supplement: Supplementary file 9 — Figure S6. Conditional depletion of Glast-creERT+ cells resulted in less severe yet “typical” HO. A&B) Gross image of HO harvested from TAM treated (A) and control (B) Nse-BMP4;Glast-creERT;ROSA26-eGFP-DTA mice after injury. Note that the gross morphology of HO in both groups was similar but the HO in the TAM treated group was smaller. Also note that a significant portion of harvest HO was not mature (without red bone marrow), which argued that quantification the immature HO with micro-CT could be misleading. C-H) Typical H&E images from treated (C, E &G) and control (D, F&H) groups both demonstrate typical features of fibro-proliferative (C&D), chondrocyte (E&F) and mature HO (G&H), though subtle differences do exist between the two groups. C-H are on the same scale, Bar = 50 μm. (TIF 18128 kb) [file 13287_2018_1107_MOESM9_ESM.tif]

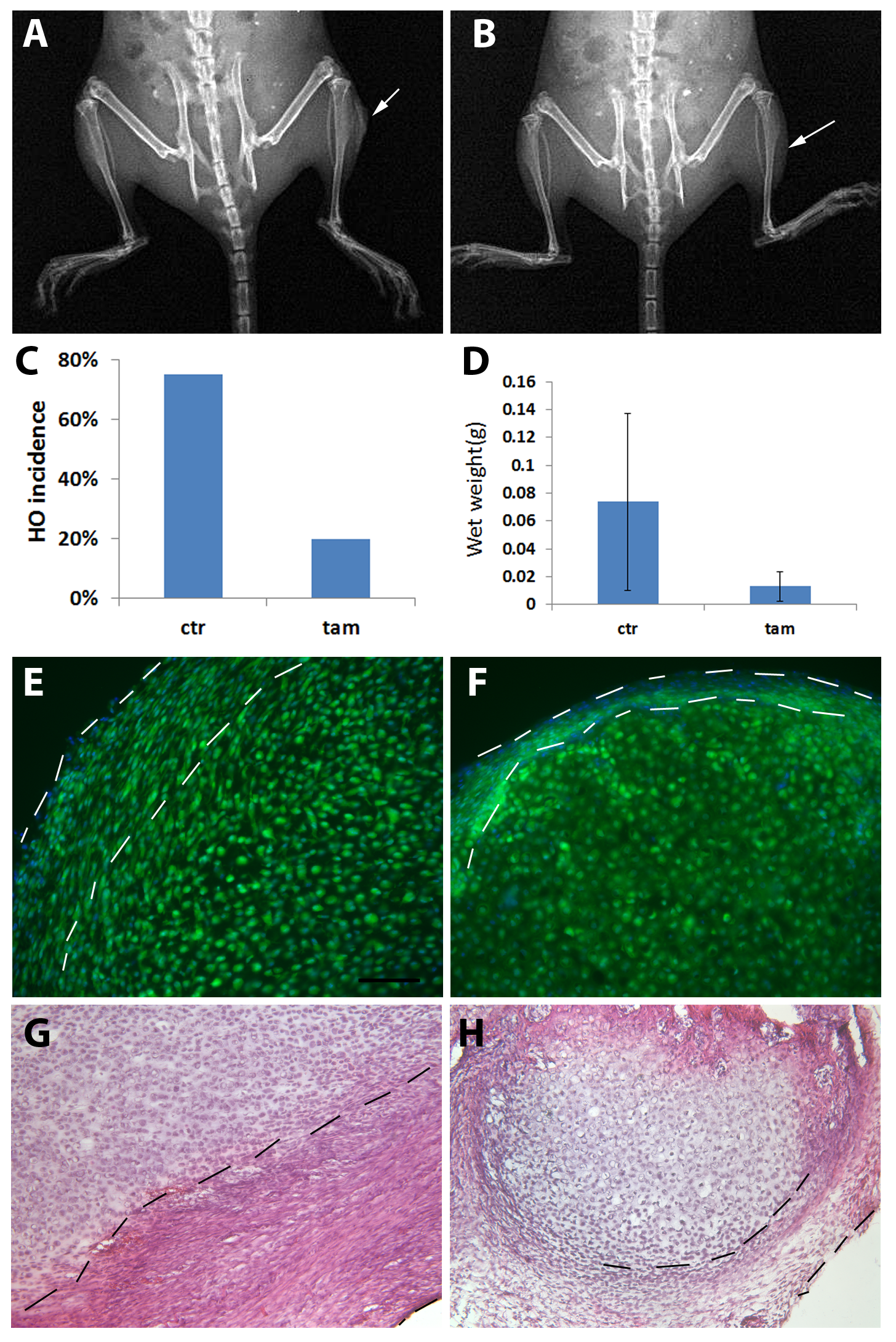

Supplement: Supplementary file 10 — Figure S7. Gli1-creERT-mediated DTA expression inhibited injury-induced HO. A&B) Typical x-ray images of control (A) and TAM treated (B) Nse-BMP4;Gli-creERT;ROSA26-eGFP-DTA mice after injury. C) HO incidence in control and TAM treated group. D) Quantification of wet weight of HO in the control and TAM treated groups. Note that depletion of Gli1-creERT-labeled cells partially inhibited but did not completely block HO. E) Typical fluorescence images from TAM treated (E) and control (F) Nse-BMP4;Gli1-creERT;ROSA26-eGFP-DTA mice. Note that in the TAM treated group (E), GFP- (recombined) cells were rarely found. G&H) H&E staining of sections from TAM treated (G) and control (H) Nse-BMP4;Gli1-creERT;ROSA26-eGFP-DTA mice. Note that both fluorescence images and H&E staining suggest that the proposed MSC domain (within dashed lines) was thinner in the TAM treated group. E-H are on the same scale, Bar = 50 μm. (TIF 15685 kb) [file 13287_2018_1107_MOESM10_ESM.tif]

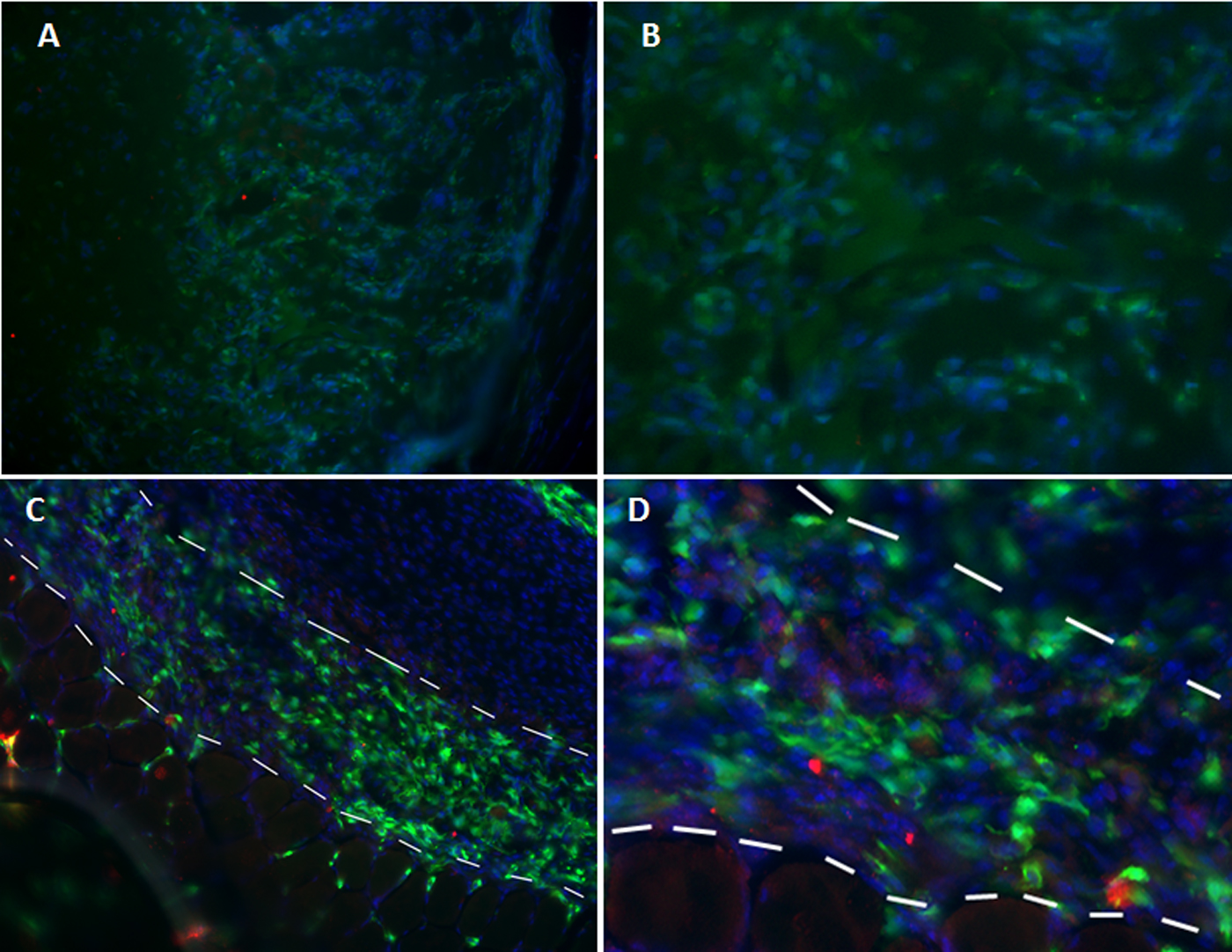

Supplement: Supplementary file 11 — Figure S8. Evidence of depletion of Gli1 in the target cells. The depletion of Gli1 in the target cells was confirmed by staining the tissue sections of Nse-BMP4;Gli1-creERT−/−;Zsgreen mice (A, low power &B, high power), and the tissues of Nse-BMP4;Gli1-creERT+/−;Zsgreen (C, low power &D, high power) mice with Gli1 antibody. Note that there is no specific staining of Gli1 (red) in the lesional tissues from Nse-BMP4;Gli1-creERT−/−;Zsgreen mice, while the specific staining of Gli1 (red) was observed in the Zsgreen+ cells in the proposed MSC niche in lesional tissues from Nse-BMP4;Gli1-creERT+/−;Zsgreen mice. (JPG 822 kb) [file 13287_2018_1107_MOESM11_ESM.jpg]

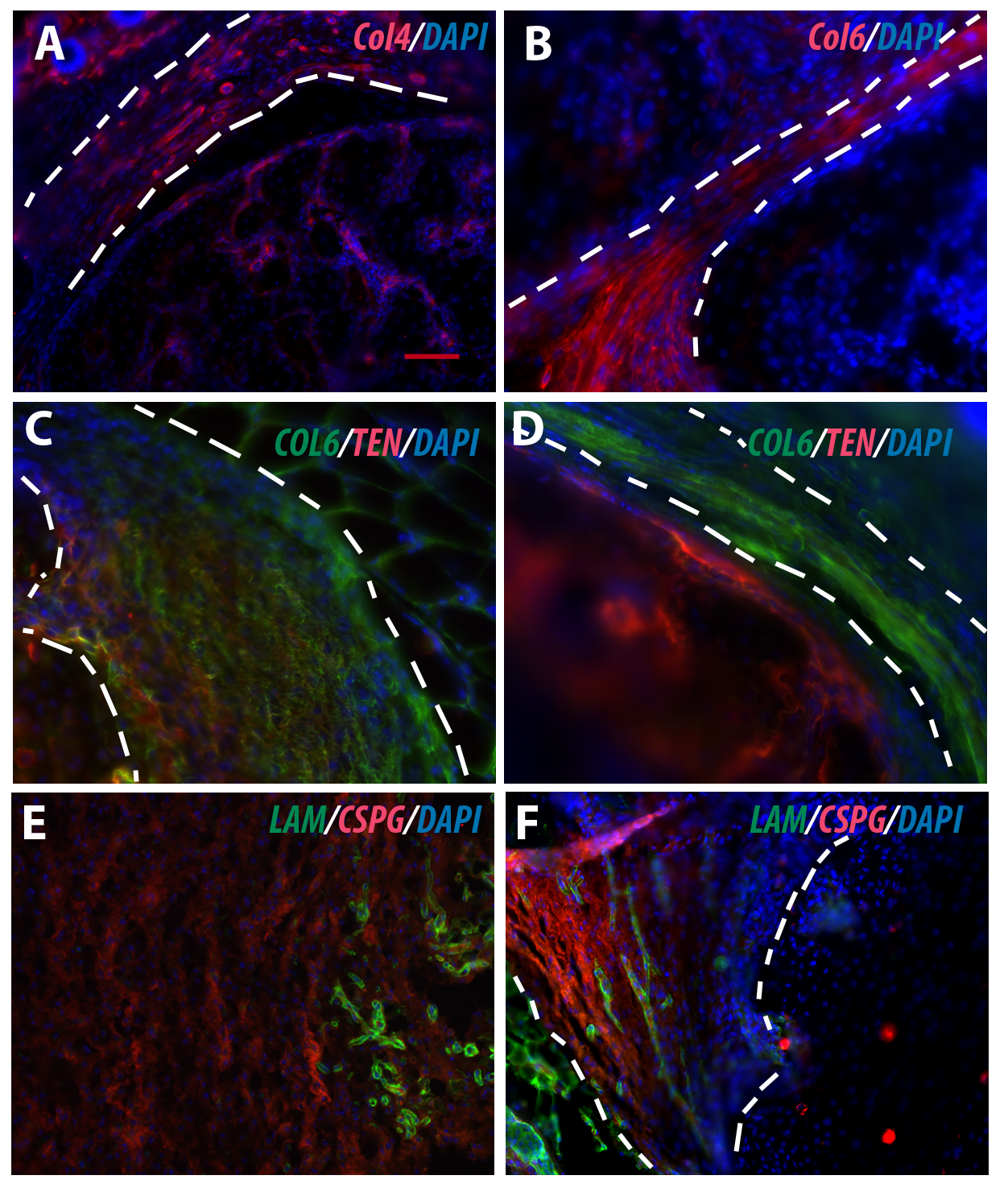

Supplement: Supplementary file 12 — Figure S9. Characterization of candidate niche supportive ECM molecules. A) Col4 was mainly involved in forming microtubular structures in the proposed MSC niche. B) Col6 was more ubiquitously upregulated in the proposed MSC niche. C) Interestingly, Col6 was closely associated with Tenascin C (TEN) only in early lesions. D) TEN was diffusedly upregulated in the early stages, but in the later stages, TEN was enriched mostly in mature domains. E&F) Similar to TEN, Chondroitin sulfate proteoglycan (CSPG) was also more or less evenly upregulated in early lesions but it became more defined as the proposed MSC niche formed. In contrast, laminin (LAM) was mainly involved in forming micro-tubular structures in the proposed MSC niche. A-F are on the same scale, Bar = 50 μm. (TIF 9818 kb) [file 13287_2018_1107_MOESM12_ESM.tif]

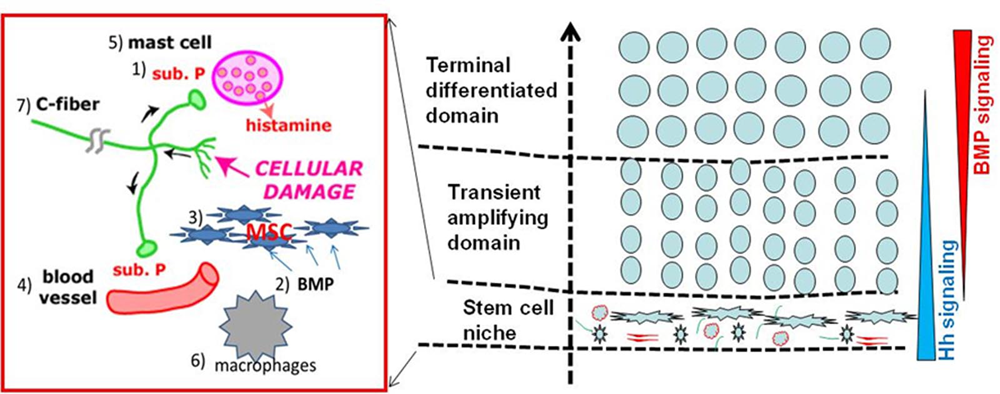

Supplement: Supplementary file 13 — Figure S10. Working model of injury-induced MSC niche. Injury-induced local microenvironment (MSC niche) is composed of niche-dwelling progenitor/stem cells (including at least Tie2-cre, Gli1-creER and Glast-creERT labeled cells) and niche supportive cells (including mast cells, neurites, vasculature and macrophages). The formation of the MSC niche likely initiates the pathological osteogenic cascade, under the co-regulation of BMP and Hh signaling through feedback and non-cell autonomous mechanisms. (TIF 1825 kb) [file 13287_2018_1107_MOESM13_ESM.tif]
