# Supplementary material for: BMP-dependent, injury-induced stem cell niche as a mechanism of heterotopic ossification
Source: Stem Cell Res Ther. 2019 Jan 11;10:14. doi: 10.1186/s13287-018-1107-7 (PMC6329163; doi:10.1186/s13287-018-1107-7)

Additional file 8

Table S3 Summary of the histomorphometric analysis of Nse-BMP4;Glast-creERT;ROSA26-eGFP-DTA mice with or without TAM treatment


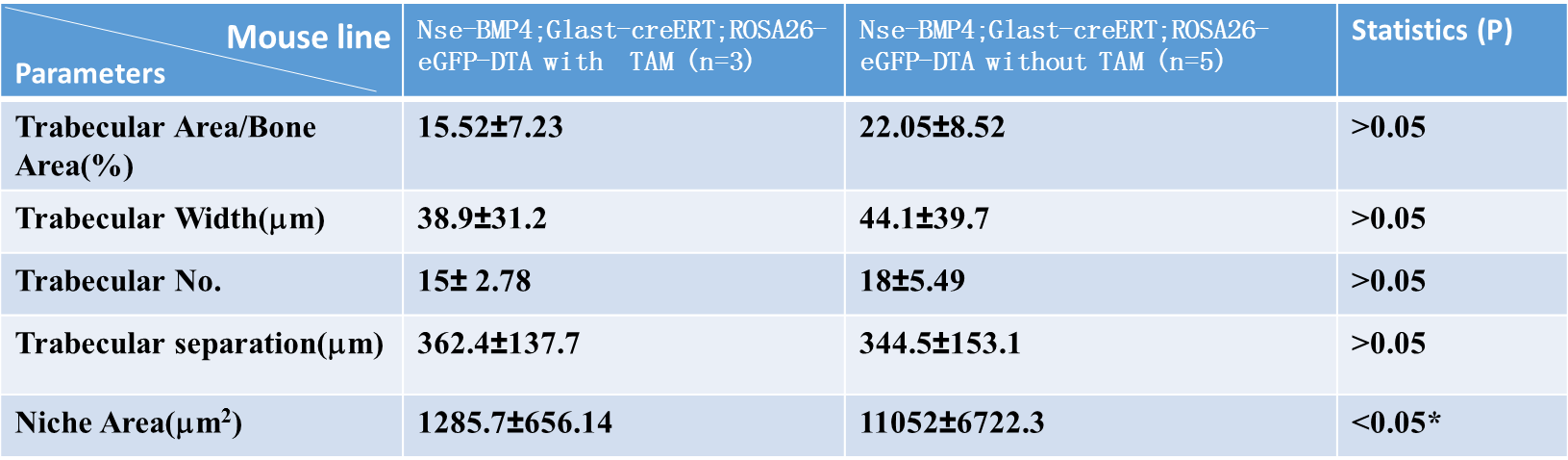

Supplement: Supplementary file 8 — Table S3. Summary of the histomorphometric analysis of Nse-BMP4;Glast-creERT;ROSA26-eGFP-DTA mice with or without TAM treatment. (DOCX 72 kb) [file 13287_2018_1107_MOESM8_ESM.docx]
